# Acclimation of *Synechocystis* sp. PCC 6803 to Alkaline pH Under Ambient Air

**DOI:** 10.1111/ppl.70474

**Published:** 2025-08-27

**Authors:** Henna Mustila, Michal Hubáček, Dorota Muth‐Pawlak, Yagut Allahverdiyeva

**Affiliations:** ^1^ Molecular Plant Biology, Department of Life Technologies University of Turku Turku Finland

**Keywords:** cyanobacteria, pH, proteomics, transporters

## Abstract

Cyanobacteria can thrive at a wide pH range from neutral up to pH 11 depending on the species. Even though cyanobacteria are alkaliphilic, only limited information on the metabolic acclimation to alkaline pH is available. In this study, we conducted a mass‐spectrometry‐based comparative proteomic analysis of *Synechocystis* sp. PCC 6803 grown in BG‐11 medium buffered at pH 7.5, pH 8.2, and pH 9.2 under ambient air. When comparing cells grown at pH 8.2 to those at pH 7.5, only minor changes in the proteome were observed, with most of the altered proteins originating from the plasma membrane. However, when comparing cells grown under pH 9.2 to those at pH 7.5, a total of 235 proteins were differentially expressed. *Synechocystis* cells growing at pH 9.2 seem to display a different composition of bicarbonate transporters. Subunits of the ATP‐dependent bicarbonate transporter BCT1 increased in abundance under pH 9.2 compared to pH 7.5 or pH 8.2, while the abundance of the sodium‐dependent bicarbonate transporter SbtA was diminished. The increased contribution of the BCT1 complex to total bicarbonate transport at high pH was confirmed by evaluating the activity of different bicarbonate transporters. Furthermore, several changes in the abundance of ion transporters were observed, including upregulation of copper, potassium, and zinc efflux proteins. In contrast, the levels of several proteins involved in nitrogen assimilation and amino acid biosynthesis were reduced at pH 9.2. To conclude, the induction of several transporters indicates the cells' ability to regulate the internal ion and pH homeostasis as well as the carbon: nitrogen ratios under alkaline conditions.

## 1 Introduction

Cyanobacteria are often considered alkaliphilic photosynthetic micro‐organisms, capable of thriving in environments ranging from neutral pH to around pH 10. Certain highly alkalitolerant cyanobacteria surviving in pH levels as high as 11 have been found in extreme alkaline environments such as soda lakes and alkaline soils (López‐Archilla et al. 2004; Singh et al. 1996). Shallow freshwater bodies are particularly prone to pH fluctuations, with high pH levels often observed during cyanobacterial blooms (López‐Archilla et al. 2004).

In aquatic environments inorganic carbon (Ci) exists in three forms: dissolved carbon dioxide (CO_2_), bicarbonate ions (HCO_3_^−^), and carbonate ions (CO_3_^2−^). In natural aquatic habitats, the bicarbonate concentration is largely dependent on the pH. In marine surface environments, the pH is typically around 8.2, and the Ci concentration remains rather stable at around 2 mM (Price et al. 2008), with the vast majority of dissolved Ci present as HCO_3_^−^, with much smaller fractions as dissolved CO_2_. In contrast, the total Ci concentration in lakes and rivers is variable, ranging from 0.1 to 4.4 mM (Assayag et al. 2006; Cole and Prairie 2014). In soda lakes, the bicarbonate concentration can reach up to 19 mM, accompanied by a high pH (Kupriyanova and Samylina 2015).

In slightly alkaline conditions (pH 7.5–pH 9), which is the typical growth range for cyanobacteria, HCO_3_^−^ ions are the dominant form of Ci (Table S1; Nguyen and Rittmann 2016). In acidic pH, the Ci equilibrium is shifted so that dissolved CO_2_ is the dominant Ci species. In highly alkaline pH (≥ pH 10), the relative abundance of Ci species shifts towards CO_3_^2−^, although the concentration of HCO_3_^−^ remains high (Mangan et al. 2016). In the current environment, with an atmospheric CO_2_ concentration of approximately 427 ppm, cyanobacteria still rely on a CO_2_ concentrating mechanism (CCM) to maintain high photosynthetic performance. The CCM operates through the active transport of bioavailable Ci species (HCO_3_^−^ and CO_2_) and subsequently accumulating a pool of HCO_3_^−^ within the cell. The import of Ci is mediated by specific bicarbonate transporters SbtA, BicA, and BCT1, as well as by the diffusion of CO_2_ into the cell. Specialized NADPH dehydrogenase (NDH) complexes, NDH‐1_3_ and NDH‐1_4_, facilitate CO_2_ uptake and convert CO_2_ to HCO_3_^−^ within the cytoplasm. Accumulating charged HCO_3_^−^ in the cytoplasm is preferred over CO_2_ because HCO_3_^−^ is substantially less permeable through the cell membranes than the uncharged CO_2_ molecule (Price 2011). The accumulated HCO_3_^−^ is further used to actively concentrate CO_2_ near the primary CO_2_ fixing enzyme, ribulose‐1,5‐bisphosphate carboxylase/oxygenase (Rubisco), which is encapsulated in protein‐shielded carboxysomes. Within the carboxysomes, carbonic anhydrase converts HCO_3_^−^ to CO_2_, ensuring a high CO_2_ concentration in the vicinity of Rubisco and thereby preventing it from using O_2_ as a substrate.

In cyanobacteria, cytosolic pH ranges between pH 7 in dark to pH 8.5 under intense light (Coleman and Colman 1981; Mangan et al. 2016). To maintain the proton motive force across the thylakoid membrane, the pH of the thylakoid lumen is typically about 2 units lower than the pH of the cytosol (Belkin et al. 1987). It has been observed that cyanobacterial internal cytoplasmic pH increases by 0.2–0.3 pH units when environmental pH increases by 2 units (Coleman and Colman 1981; Jiang et al. 2013; Ritchie 1991). While thylakoid lumen pH is higher in the dark than in light, it responds negligibly to changes in external pH within the range of 7.0–8.0 (Belkin et al. 1987). Hence, maintaining pH gradients across the membrane systems is crucial for cyanobacterial growth, especially upon changes in environmental pH.

Although cyanobacteria are recognized for their alkaliphilic nature, most research involving the model cyanobacterium *Synechocystis* sp. PCC 6803 (hereafter *Synechocystis*) has been conducted with growth media buffered around pH 7.5–8. Transcriptomic data have provided insight into the initial response of *Synechocystis* cells to alkaline pH after a few hours shift from pH 7.5 to pH 10 (Summerfield et al. 2013; Summerfield and Sherman 2008) and with the same pH range, but under a 3% CO_2_ atmosphere (Billis et al. 2014). Among the main findings were upregulation of certain Na^+^/H^+^ antiporters, ion transporters, amino acid deaminases, and ATP synthase in response to elevated pH, which are in agreement with mechanisms detected in other bacteria (Krulwich et al. 2011). The function of Na^+^/H^+^ antiporters was shown to be important not only for salt tolerance but also for pH homeostasis, particularly in alkaline conditions in cyanobacteria (Billini et al. 2008; Elanskaya et al. 2002; Tsujii et al. 2025). Moreover, upregulation of genes involved in cell wall biosynthesis and signal transduction has been detected in *Synechococcus* sp. PCC 7942 in response to a shift to high pH (Billis et al. 2014). There is also evidence that environmental pH, and thus the periplasmic pH, might affect the PSII biogenesis via thylakoid centers, where thylakoid and cytoplasmic membranes are in close contact (Morris et al. 2016). Furthermore, gel‐based proteomics data have elucidated the importance of plasma membrane and periplasmic proteins for the pH response in *Synechocystis* (Kurian et al. 2006; Zhang et al. 2009). However, to the best of our knowledge, comparative proteomic profiling of cyanobacteria under high pH conditions, which could provide insights into metabolic rearrangements, is still lacking.

Considering that pH is tightly linked to Ci availability, several studies have attempted to decouple these two parameters. Nguyen and Rittmann (2016) showed that the highest specific growth rate of *Synechocystis* was observed at pH 8.5, compared to pH 7.5 and pH 9.5, when comparable amounts of bioavailable Ci were maintained by adding NaHCO_3_ to reach concentrations between 0.3 and 3.4 mM. Their findings indicated that growth was saturated at about 1 mM bioavailable Ci. In another study, *Synechocystis* growth was monitored up to pH 11 in the presence of 3% CO_2_ (Touloupakis et al. 2016). Biomass productivity and biochemical composition remained relatively stable between pH 7 and 10, and photosynthetic activity remained constant up to pH 11. These studies highlight the remarkable capacity of cyanobacteria to acclimate to very high pH conditions when supplied with sufficient bioavailable Ci. However, careful and independent control of pH and Ci is rarely achieved in natural or standard laboratory conditions. In aquatic habitats, high pH typically coincides with high bicarbonate alkalinity. Therefore, the physiological state and proteomic responses of cyanobacterial cells in different pH ranges remain to be elucidated.

In this study, we performed a physiological characterization and comparative proteomic analysis of *Synechocystis* to investigate the molecular acclimation responses of cyanobacterial cells grown under near‐neutral (pH 7.5) and alkaline (pH 8.2 and pH 9.2) conditions. To monitor pH acclimation, we used buffered BG‐11 medium to maintain a stable pH, while allowing the Ci in the liquid medium to equilibrate with ambient air. Using label‐free liquid chromatography–tandem mass spectrometry (LC–MS/MS) for quantitative proteomic analysis, we identified more than 2000 proteins. The results revealed significant modifications in plasma membrane proteins at pH 8.2 and extensive changes in the whole proteome at pH 9.2, compared to cells grown at neutral pH. Notably, proteins with increased abundance at pH 9.2 included subunits of the BCT1 bicarbonate transporter complex. We further examined the activity of this transporter in comparison to the two other known bicarbonate transporters in *Synechocystis* under various pH conditions.

## 2 Material and Methods

### 2.1 Growth Conditions

The glucose‐tolerant variant of *Synechocystis* sp. PCC 6803 was used in the present study. Cells were grown at 30°C in atmospheric air under constant illumination of 50 μmol photons m^−2^ s^−1^ with LED white light. Cultures (30 mL) were grown in 100‐mL Erlenmeyer flasks, agitated at 120 rpm in an AlgaeTron AG 230 growth chamber (PSI Instruments). BG‐11 medium was buffered with 20 mM HEPES (pH 7.5), 20 mM TES‐KOH (pH 8.2), or 20 mM N‐Cyclohexyl‐2‐aminoethanesulfonic acid (CHES, pH 9.2 or pH 10.0), depending on the pH condition. Preexperimental cultures were grown for four days in 30 mL of BG‐11 medium buffered to each of the three pH regimes. Experimental cultures were then inoculated at OD_750_ = 0.1 in BG‐11 medium supplemented with the buffer corresponding to the preexperimental culture. For proteomics analysis, the cultures were grown under the experimental conditions until reaching OD_750_ ≈ 0.9. This process took 70, 84, and 85 h for cultures grown at pH 9.2, pH 8.2, and pH 7.5, respectively. For chlorophyll *a* (Chl *a*) determination, room temperature absorption spectra, and Membrane Inlet Mass Spectrometry (MIMS) analysis, cells were grown for three days.

### 2.2 Analytical Methods

Room temperature absorption spectra were recorded as described in Mustila et al. (2021). Chl *a* was extracted from cells using 90% methanol, and its concentration was assessed based on absorbance at 665 nm (Meeks and Castenholz 1971). Cell count was determined with a Cellometer Auto X4 (Nexcelom Bioscience). Samples were adjusted to OD_750_ = 0.2, brightfield images were captured, and the cell number was determined by the Nexcelom software.

### 2.3 Mass Spectrometry Data‐Dependent Acquisition

For a liquid chromatography–tandem mass spectrometry (LC‐ESI‐MS/MS) analysis, total proteins from *Synechocystis* were isolated and digested as described previously (Mustila et al. 2021).

The LC‐ESI‐MS/MS analyses were performed on a nanoflow HPLC system (Easy‐nLC1200, Thermo Fisher Scientific) coupled to the Q Exactive HF mass spectrometer (Thermo Fisher Scientific) equipped with a nano‐electrospray ionization source. Extracted peptides equivalent to 400 ng from four biological replicates per condition were loaded in a randomized order onto the nLC column. An in‐house packed 40 cm, 75 μm ID capillary column with 1.9 μm Reprosil‐Pur C18 beads (Dr. Maisch, Ammerbuch) was used. The column temperature was maintained at 60°C using an in‐house‐made column oven and interfaced online with the mass spectrometer. The mobile phase consisted of water with 0.1% formic acid (solvent A) or acetonitrile/water (80:20 (v/v)) with 0.1% formic acid (solvent B). The MS data were acquired with the Thermo Xcalibur 3.1 software (Thermo Fisher Scientific).

The protein identification and label‐free data‐dependent acquisition (DDA) method consisted of an Orbitrap MS survey scan with the mass range of 300–1800 m/z, followed by HCD fragmentation for the 10 most intense ions with charge ≥ 2. Spectra were detected with a resolution of 120,000 and 15,000 (at m/z 200) for full scan and for fragment ions, respectively, and the HCD scan used a normalized collision energy of 27%. The automatic gain control (AGC) was set to 3e6 and 1e5 ions, and the maximum ion injection time was 100 and 250 ms for the MS and MS/MS scans, respectively.

### 2.4 Proteomics Data Analysis

Proteins in the raw files were identified according to the *Synechocystis* database retrieved from Cyanobase (Kaneko et al. 1996; 3672 entries, 23.10.2012) using the Proteome Discoverer 2.5 software (Thermo Fisher Scientific) connected to an in‐house server running the Mascot 2.6.1 (Perkins et al. 1999) search engine (Matrix Science). Precursor mass tolerance value was restricted to monoisotopic mass ±10 ppm and fragment ion to ±0.02 Da. For the validation of the identified spectra, the Percolator (Käll et al. 2007) algorithm was used with a relaxed false discovery rate (FDR) of 0.05. The mass spectrometry data and protein identification files data have been deposited to the ProteomeXchange Consortium via the PRIDE (Perez‐Riverol et al. 2025) partner repository with the dataset identifier PXD061233 and https://doi.org/10.6019/PXD061233. Label‐free DDA quantification was conducted using the Progenesis (Waters) software with global normalization and using relative quantification of proteins with at least two unique peptides per protein. The statistical test analysis of variance (ANOVA) was used, and the statistical significance threshold was set to a *p* value ≤ 0.05. The threshold of Log_2_ fold change (FC) was set to 0.58 for more abundant and −0.58 for less abundant proteins. Venn diagrams were made with a web‐based tool (https://www.interactivenn.net/; Heberle et al. 2015).

### 2.5 Membrane Inlet Mass Spectrometry

The in vivo gas fluxes of ^16^O_2_ (m/z = 32), ^18^O_2_ (m/z = 36), and CO_2_ (m/z = 44) were measured by an in‐house built MIMS as described earlier (Mustila et al. 2016). For assessment of the HCO_3_^−^ consumption rate, the cells were washed and then resuspended in modified BG‐11 medium without added NaNO_3_ or Na_2_CO_2_ and supplemented with 20 mM CHES‐KOH pH 10.0. The cells were adjusted to 10 μg Chl *a* mL^−1^ and kept at 50 μmol photons m^−2^ s^−1^ at 30°C. Before the measurements, cells were supplemented with ^18^O_2_ at an equivalent concentration to ^16^O_2_ and with 1.5 mM KHCO_3_ to provide sufficient Ci source without adding Na^+^. When indicated, cells were also supplemented with 25 mM NaCl or with 25 mM LiCl. Gas exchange was monitored over a 5‐min dark period, followed by a 5‐min illumination period under 500 μmol photons m^−2^ s^−1^ of white actinic light, and then another 5‐min dark period. The gas exchange rates were calculated as in Beckmann et al. (2009). The gross O_2_ evolution rates were calculated as an average between 3.5 and 4.5 min under illumination.

### 2.6 Fluorescence Measurements

The Chl *a* fluorescence from intact cells was recorded with a pulse amplitude‐modulated spectrophotometer (Dual‐PAM‐100; Walz). Harvested cells were resuspended in fresh BG‐11 pH 7.5 and adjusted to 10 μg Chl *a* mL^−1^. Before the measurements, cells were dark‐adapted for 15 min. Then, 50 μmol photons m^−2^ s^−1^ was applied as actinic light. Saturating pulses of 5000 μmol photons m^−2^ s^−1^ (300 ms) and strong far‐red light (720 nm, 75 W m^−2^) were applied to the samples when required. Fm^D^ was recorded upon the first saturating pulse, following darkness. Fm^FR^ was recorded by applying a saturating pulse at the end of 8 s of strong far‐red illumination, and the maximal fluorescence during illumination (Fm′) was recorded upon a saturating pulse during illumination.

Flash‐induced fluorescence yield and subsequent relaxation was monitored using a fluorometer (FL3500, PSI Instruments). Prior to the measurement, cells were adjusted to 5 μg Chl *a* mL^−1^ and dark adapted for 5 min. When indicated, fluorescence relaxation was measured in the presence of 20 μM DCMU.

## 3 Results and Discussion

### 3.1 Characterisation of *Synechocystis* at High pH

**FIGURE 1:**
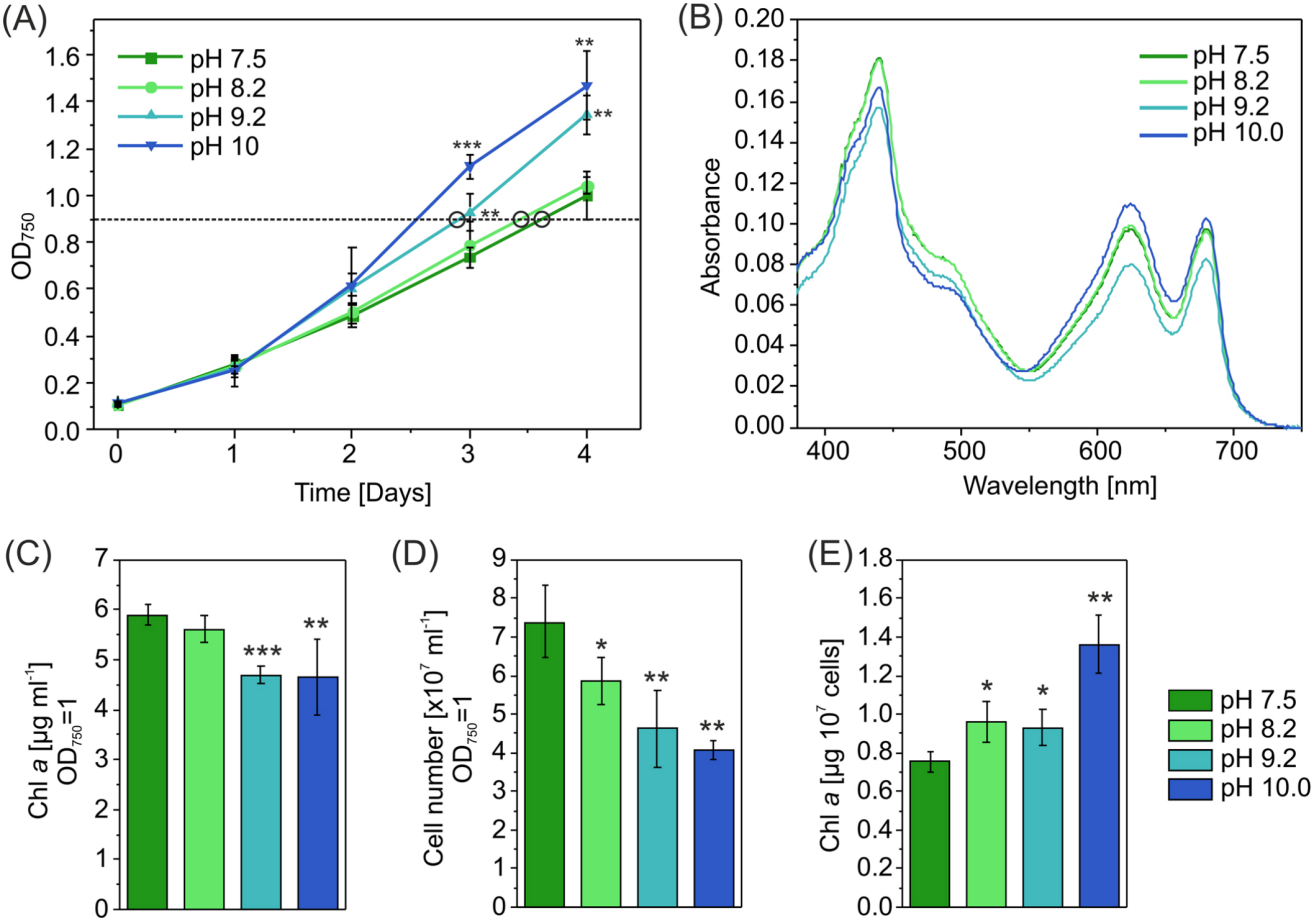
Characterization of *Synechocystis* growth and pigment content under different pH conditions. (A) Growth of *Synechocystis* measured by OD_750_. The time point for collection of cells for proteomics (OD_750_ = 0.9) is indicated with black circles. (B) Room temperature absorbance spectra, (C) Chl *a* content, (D) number of cells in 1 mL at the OD_750_ of 1 and (E) the Chl *a* amount per 10^7^ cells were determined after three days of cultivation at the corresponding pH. Absorbance spectra represent the average of three biological replicates. Error bars show standard deviations from 3 to 4 biologically independent experiments. The asterisks represent significant differences between cells cultivated in medium with pH 7.5 and higher pH, as determined by Student's t test (*p < 0.05, **p < 0.01, ***p < 0.001).

To investigate the long‐term response of *Synechocystis* to different pH levels, cells were cultivated in media at pH 7.5, 8.2, and 9.2 for four days. After the acclimation, experimental cultures were inoculated at OD_750_ = 0.1 in BG‐11 medium buffered to match the preexperimental pH. The cultures were grown under 50 μmol photons m^−2^ s^−1^ continuous light to OD_750_ ≈ 0.9 and then harvested for the proteomics analysis (Figure 1A). Despite using buffered BG‐11 medium to maintain specific pHs, moderate pH shifts were observed during growth (Table S2). For the comparative proteomic analysis, we selected pH conditions commonly used in laboratory settings. In addition to these three pH conditions, *Synechocystis* growth was also assessed at pH 10.0. Growth rates were similar between cultures buffered at pH 7.5 and pH 8.2, while growth was enhanced at pH 9.2 and pH 10.0, as measured by OD_750_. This observation is in line with previous studies (Chaves et al. 2015; Kurian et al. 2006), which show that alkaline conditions favor photoautotrophic growth in *Synechocystis*, at least under moderate light conditions. It is noteworthy that, under these conditions, cell growth is likely constrained by both light availability and Ci. Although the bicarbonate concentration in the cell‐free medium increases with pH (Table S1), it is continuously consumed by the cells during growth (Carrasquer‐Alvarez et al. 2025). As total Ci was not measured in the cell‐containing cultures, and the actual concentration of dissolved Ci depends on the rate of cellular uptake, the precise bicarbonate concentration encountered by the cells under each pH condition remains undetermined.

The total protein yield extracted from cells did not differ significantly between samples grown at different pH levels (Figure S1). However, despite enhanced growth at alkaline pH, the Chl *a* content of cells grown at pH 9.2 or pH 10.0 was lower compared to those cultivated at pH 7.5 after three days of cultivation (Figure 1B,C) and again after four days (Figure S2A). Similarly, carotenoid content was lower at pH 9.2 and 10.0 (Figure 1B). As carotenoids function as protective pigments, their lower accumulation may suggest that cells grown at high pH experience reduced stress. To gain a more detailed view of cell physiology, we also determined the cell number at various pH levels, showing a lower cell count per OD at higher pH (Figures 1D and S2B). Hence, the Chl *a* content per cell increased with higher pH (Figures 1E and S2C). Lower cell count may indicate bigger cell size; however, no detectable differences in cell size were observed under the varying pH conditions (Table S3). It is also possible that altered pigment or glycogen content could affect light scattering and consequently, influence OD_750_ measurements. Identifying the underlying cause would require more detailed investigations.

### 3.2 DDA for Proteomic Profiling of *Synechocystis*

**FIGURE 2:**
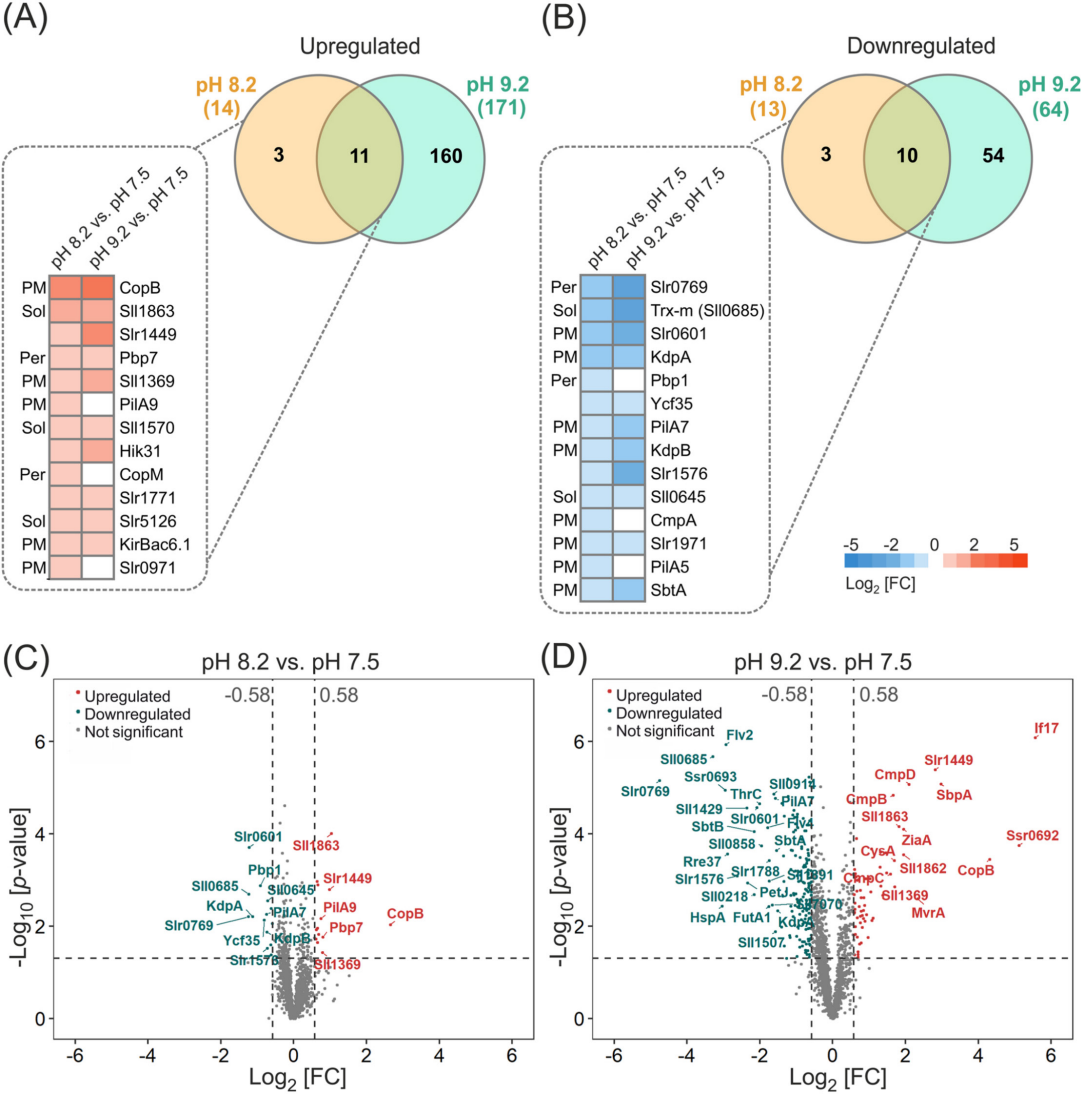
Protein abundance analysis under alkaline pH. (A, B) Venn diagram showing the differentially upregulated (A) and downregulated (B) proteins that are commonly or uniquely detected in each comparison. Heatmap specifies the up‐ and downregulated proteins at pH 8.2 and their corresponding change at pH 9.2. Localization of the shown proteins are indicated; Sol: soluble proteins, PM: plasma membrane and Per: periplasm. (C, D) Volcano plot displaying the extent and statistical significance of protein abundance alterations. The differential expression of proteins in (C) Log_2_FC of pH 8.2 versus pH 7.5 and (D) Log_2_FC of pH 9.2 versus pH 7.5. The −log_10_ (*p*‐value) is plotted against the Log_2_FC. The two vertical dashed lines denote ±0.58 Log_2_FC while the dashed horizontal line shows *p* = 0.05 significance threshold. The most significantly altered proteins are highlighted with their gene names, red indicating upregulated proteins and blue indicating downregulated proteins.

Using a label‐free MS‐based quantitative protein analysis, we identified 2049 proteins with at least two peptides for each, accounting for approximately 56% of the predicted *Synechocystis* proteome (Table S4). Among these, 2019 proteins were quantified across all samples by label‐free DDA mass spectrometry (Table S5). In cells grown at pH 8.2 and pH 9.2, 27 and 235 proteins, respectively, showed a differential expression compared to the control (pH 7.5; Figure 2A,B). The volcano plot shows the distribution of up‐ and downregulated proteins in pH 8.2 (Figure 2C) and pH 9.2 (Figure 2D) compared to pH 7.5.

**FIGURE 3:**
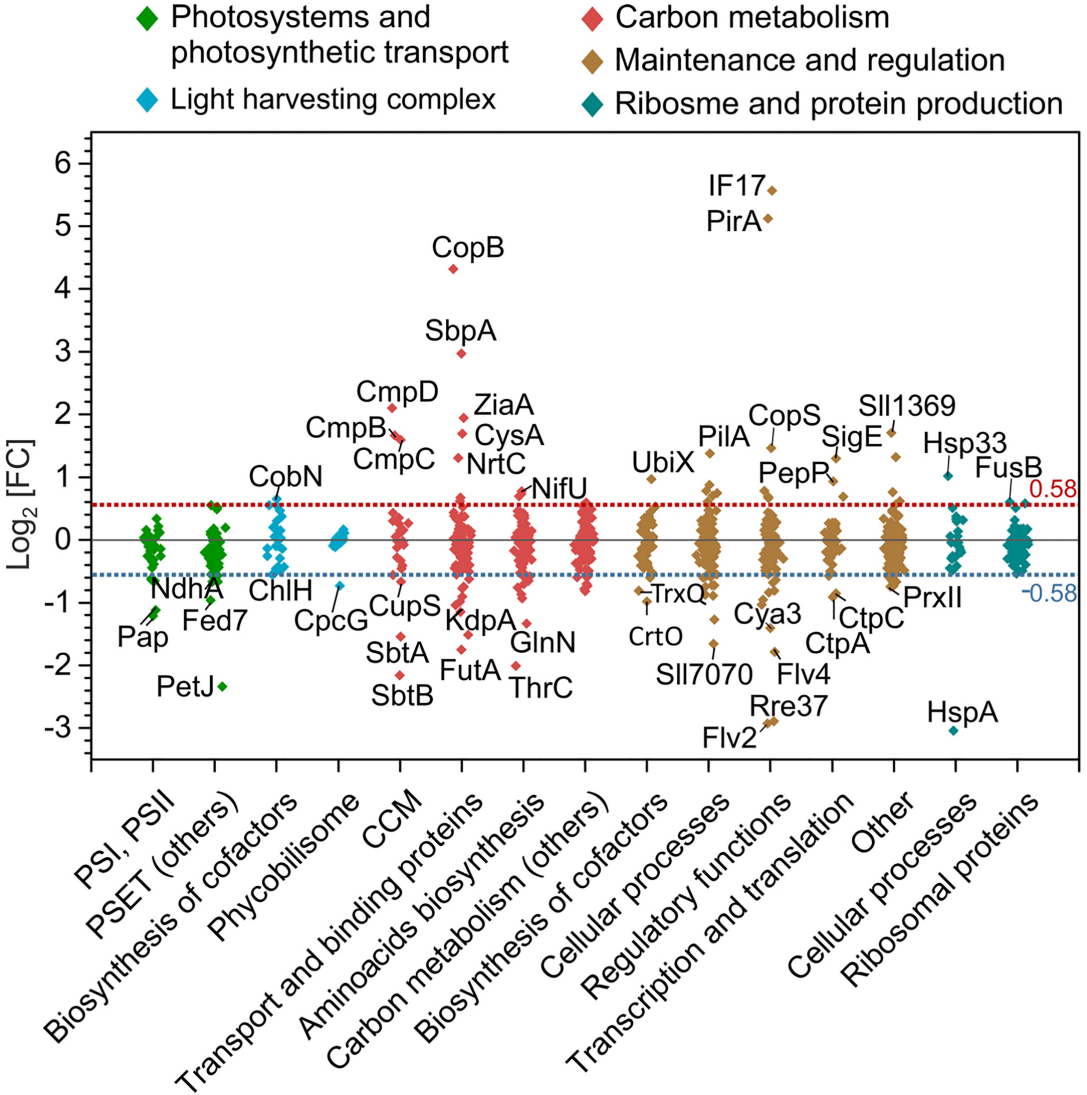
Differentially expressed proteins at alkaline pH. Influence of the pH on basic cellular processes (pH 9.2 vs. pH 7.5) divided in selected categories according to (Jahn et al. 2018).

Proteins quantified and compared between pH treatments were classified into functional categories following the main and subcategories defined by Jahn et al. (2018). Five main categories are shown in Figure 3 (Table S6). The highest fraction of differentially expressed proteins was found in the categories of carbon metabolism (including CCM, transport and binding proteins, and amino acid biosynthesis) and maintenance and regulation (regulatory functions).

### 3.3 Global Evaluation of the Proteome at Alkaline pH

#### 3.3.1 Transporter Proteins

**FIGURE 4:**
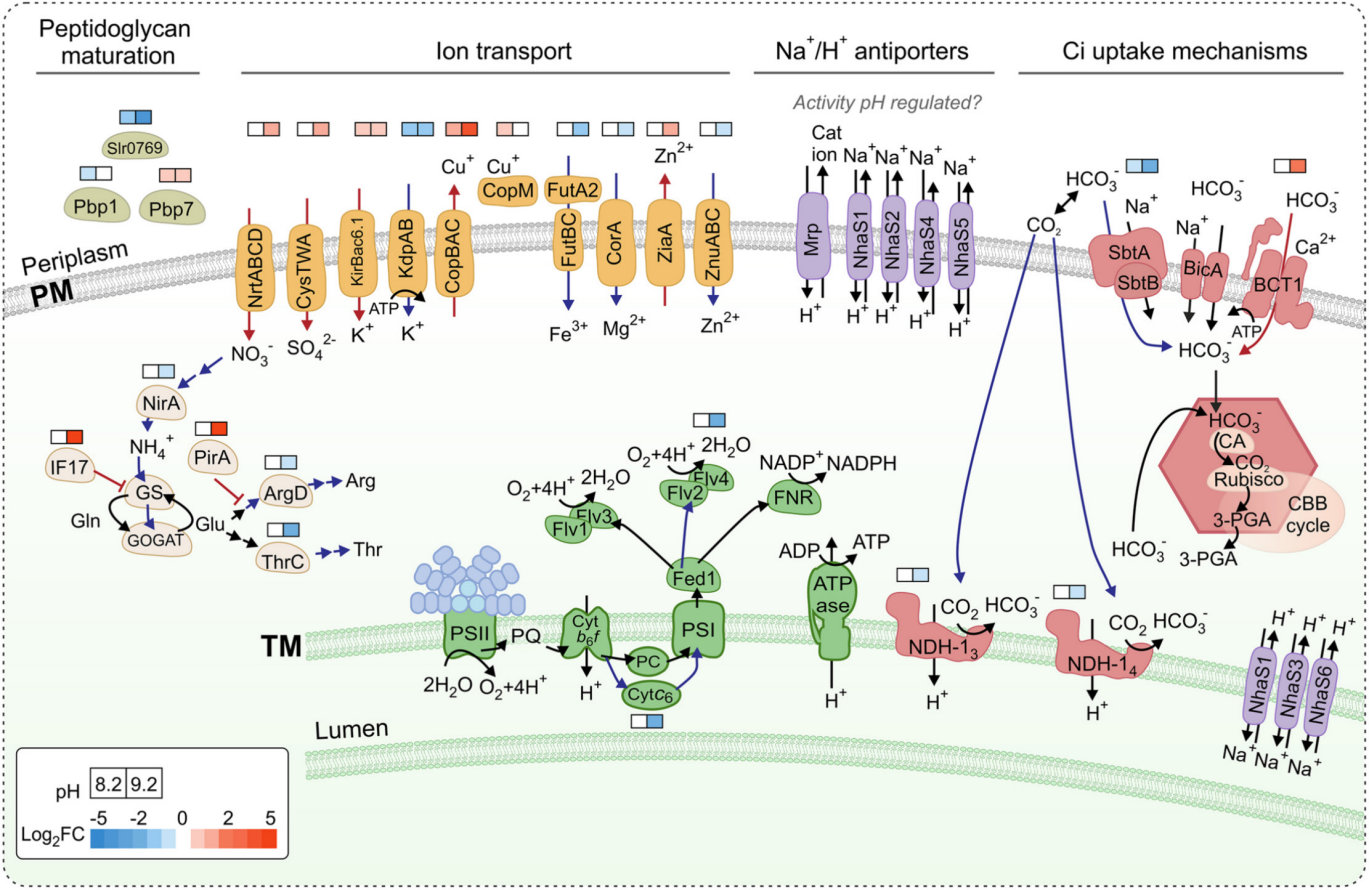
Schematic representation of the regulation of metabolism in *Synechocystis* sp. PCC 6803 cells grown under alkaline pH. Differential protein expression (squares) is shown above the name of the protein reflecting the degree of down‐ (blue) or upregulation (red) at pH 8.2 or pH 9.2 in comparison to pH 7.5. More abundant proteins and pathways are marked with red, and less abundant proteins and routes are marked with blue. PM, plasma membrane; TM, thylakoid membrane.

Nearly half (13 proteins) of the proteins that show significant changes during growth at pH 8.2, compared with pH 7.5, are located in the plasma membrane (Figure 2A,B). In addition, four of these proteins are predicted to be located in the periplasm. The impact of pH on these proteins emphasizes the importance of the cyanobacterial plasma membrane in maintaining cytoplasmic integrity and mediating interactions with the external medium. The majority of these proteins show a similar abundance in pH 9.2, although growth in a more alkaline pH evokes widespread alterations in the entire *Synechocystis* proteome. Many of the differentially expressed membrane proteins are metal and ion transporters. The abundance of sulfate uptake proteins (CysA, SbpA) and a zinc efflux protein (ZiaA, a putative Zn‐efflux PI‐type ATPase) increased, while proteins involved in zinc uptake (ZnuA) and magnesium transport (CorA) were diminished (Figure 4). It is worth noting, that many transporters may exhibit affinity to more than one metal ion and the substrate specificity should not be evaluated solely based on sequence homology. Nonetheless, the function of transporters presented here are based on the current literature. For example, Cu^+^ has been shown to displace Zn^2+^ from the metal binding domain of ZiaA (Badarau and Dennison 2011). At pH 9.2, two subunits of the putative K^+^‐transporting P‐type ATPase, KdpA and KdpB (Nanatani et al. 2015), were less abundant, whereas the low‐affinity K^+^‐transporter, KirBac6.1 (Slr5078), showed an increased abundance (Paynter et al. 2010). In addition, two ATP‐binding cassette (ABC) transporter proteins (Slr0615, Slr0864) with unknown substrates were differentially regulated. Furthermore, the outer membrane iron ABC transporter FutA1 and the periplasmic iron‐binding protein FutA2 were less abundant at pH 9.2. The FutA2 has also been shown to play a role in copper import (Waldron et al. 2007).

Antiporters involved in alkaline homeostasis in many bacteria catalyze the extrusion of Na^+^ or K^+^ from the cells in exchange for H^+^ influx, leading to cytoplasmic acidification (Krulwich et al. 2011). *Synechocystis* has six Na^+^/H^+^ antiporters (NhaS1 to NhaS6), of which NhaS1, NhaS2, NhaS4, and NhaS5 are located in the plasma membrane, while the other two reside on the thylakoid membrane (Tsujii et al. 2025). However, Na^+^/H^+^ or K^+^/ H^+^ antiporters did not exhibit major changes when grown at alkaline pH. Among cation/H^+^ antiporters, only the amount of MrpA was slightly enhanced (Log_2_FC = 0.41, *p* = 0.049) at pH 9.2, while other subunits of the Mrp (Multiple resistance and pH or Mnh) antiporter system were not detected in this study. The hetero‐oligomeric Na^+^/H^+^ antiporter Mrp has been shown to be important for pH homeostasis in alkaline environments for various heterotrophic bacteria (Ito et al. 2017; Krulwich et al. 2011) as well as in *Anabaena* sp. PCC7120 (Blanco‐Rivero et al. 2005). In addition, cytoplasmic Ca^2+^ levels have been linked to intracellular pH regulation in bacteria (Jiang et al. 2013; O'Hara et al. 1989) and plant cells (Behera et al. 2018). Summerfield and Sherman (2008) reported increased transcript levels of *nhaS3* (Na^+^/H^+^ antiporter) and *mrp*‐operon genes in *Synechocystis* following a shift to pH 10.0. However, (Billis et al. 2014) found no significant changes for cation/H^+^ antiporter transcripts after a 24 h shift from pH 7.5 to pH 10.0 under 3% CO_2_. Altogether, these data suggest that *Synechocystis* does not drastically alter its cation/H^+^ antiporter profile at moderately high pH but instead maintains relatively stable protein levels. This constitutive expression may represent an adaptive strategy for coping with rapid pH changes regularly experienced by *Synechocystis* and other alkalophilic cyanobacteria in their natural habitats. Furthermore, the cation/H^+^ antiporter activity may be regulated posttranslationally in a pH‐dependent manner, as demonstrated for the *E. coli* NhaA Na^+^/H^+^ antiporter (Taglicht et al. 1991). Therefore, while NhaS antiporter protein levels remain unchanged, their activity may dynamically adjust in response to external pH.

CopS, part of the CopRS two‐component system (also known as Hik31‐Rre34) was found in higher abundance at pH 9.2. The *Synechocystis* genome harbors two copies of the *copMRS* operon; one copy is located in the chromosome and the other in the native plasmid pSYSX. The gene copies are almost identical, since the proteins encoded from these duplicated genes share 95%–98% identity of the amino acid sequences. With the DDA method used here, we cannot distinguish whether the corresponding transcripts for the CopMRS proteins are encoded from the plasmid or from the chromosome. Intriguingly, the plasmid *copMRS* operon was shown to mediate alkaline pH homeostasis in the presence of glucose, and the Hik31‐Rre34 system is involved in integrating photosynthetic, nitrogen, and carbon metabolism (Nagarajan et al. 2014). It has also been shown that the *copMRS* operon is induced by several conditions that alter the redox state of the cell in a copper‐dependent manner (Giner‐Lamia et al. 2012; Hihara et al. 2003; López‐Maury et al. 2012). In accordance, the amounts of CopB and CopC proteins, which are part of the plasma membrane‐localized CopBAC complex responsible for Cu^2+^ efflux (Giner‐Lamia et al. 2012), were enhanced at pH 9.2. In fact, CopB, Hik31, and a copper‐binding protein CopM showed a significant upregulation also at pH 8.2 in comparison to pH 7.5 (Figure 3). Besides the response to copper, the enhanced abundance of CopBAC could be explained by the interdependence of copper and iron homeostasis (Bernal et al. 2012) or a hypothetical function of CopBAC as a low‐affinity Mn exporter (Reis et al. 2024).

In *Synechocystis*, either plastocyanin (PetE) or cytochrome *c*_6_ (PetJ) may function as an electron carrier between cytochrome *b*_6_ *f* and PSI depending on the copper availability. Under copper depletion, plastocyanin is replaced by a haem‐containing cytochrome *c*_6_. Here, cytochrome *c*_6_ was found repressed at high pH, while plastocyanin showed a slight increase (Log_2_FC = 0.55, *p* = 0.004). Along with cytochrome *c*_*6*_, a small protein with an unknown function, Slr0601, was significantly reduced in its abundance. The expression of *petE*, *petJ*, and *slr0601*‐*slr0602* is regulated by the PetRP system (García‐Cañas et al. 2021). Overall, it is unlikely that the Cop complex, the CopRS two‐component system, and the switch from cytochrome *c*_6_ to plastocyanin show a specific pH response. Instead, they appear to be connected to the redox status of the photosynthetic electron transport chain, contributing to a general stress response in *Synechocystis*.

#### 3.3.2 Photosynthetic Proteins

Among the proteins involved in photosynthetic pathways, six proteins, Slr0144‐Slr0147, Slr0149, and Slr0151, encoded by the genes in a Pap operon (Photosystem II Assembly Proteins), were found to be less abundant at pH 9.2. Pap proteins play an important role in the assembly of PSII and PSI (Kubota et al. 2010; Wegener et al. 2008). Additionally, a phycobilisome “rod‐core linker” protein, CpcG2, was also less abundant at pH 9.2. Unlike typical phycobilisomes containing CpcG1, which mainly transfer light energy to PSII (Kondo et al. 2007), those with CpcG2 lack the allophycocyanin core and preferentially transfer energy to PSI. The two‐component system histidine kinase CcaS (Hik32) and the response regulator CcaR (Slr1584; Hirose et al. 2008) which regulate the expression of CpcG2, were also diminished. Even though the Chl *a* content per cell was slightly higher at pH 9.2, compared to cells grown at pH 7.5 (Figure 1E), no significant differential expression was detected for the core proteins of PSI or PSII. Although, one Chl *a* biosynthesis protein, ChlH, showed a slight downregulation (Log_2_FC = −0.53, *p* = 0.04). The lower abundance of CpcG2 and ChlH as well as assembly factors encoded by the Pap operon might indicate that less light energy is required for the distribution to PSI under alkaline conditions.

A strong decrease in flavodiiron proteins Flv2 and Flv4 was observed at pH 9.2 compared to pH 8.2 or pH 7.5 (Figure 2D), which is in agreement with our previous findings (Santana‐Sanchez et al. 2019). Flv2/Flv4 hetero‐oligomers catalyze a steady light‐driven O_2_ photoreduction downstream of PSI (Santana‐Sanchez et al. 2019). Their downregulation at high pH might contribute to cytoplasmic proton retention, as the Flvs introduce protons to oxygen.

#### 3.3.3 Protein Expression, Modifications, and Stress Response

Among the transcription‐related proteins identified at high pH, the redox‐regulated RNA helicase CrhR (Slr0083) was upregulated. CrhR is believed to regulate the expression of redox‐responsive genes at the translational level (Kujat and Owttrim 2000; Ritter et al. 2020), and a role has been implicated in multiple abiotic stress responses (Klähn et al. 2021; Ritter et al. 2020). Intriguingly, the ribosomal protein S12 methylthiotransferase RimO (Slr0082), which is transcribed from the same operon as CrhR, showed reduced abundance. This suggests a role for posttranscriptional regulation, probably mediated by asRNA, as shown by Klähn et al. (2021). Furthermore, the translation elongation factor G2 (FusB, Sll1098) was more abundant at pH 9.2, indicating broader changes in protein synthesis under alkaline conditions.

Two carboxyl‐terminal proteases, CtpA and CtpC, showed lower abundance, whereas the D‐alanyl‐D‐alanine carboxypeptidase (Pbp7, Slr1924) accumulated more at pH 9.2. The CtpA is responsible for the proteolytic removal of a small extension at the carboxyl‐terminus of a precursor form of D1 during the maturation process (Satoh and Yamamoto 2007). The CtpC and Pbp7 have been isolated from the periplasmic fraction and therefore they could be involved in the processing of periplasmic or cell wall‐associated proteins (Fulda et al. 2000). Indeed, the Pbp7 has been implicated in peptidoglycan biosynthesis, which is a key component of the cell wall (Marbouty et al. 2009).

Three oxidative stress proteins (type II peroxiredoxin PrxII, thioredoxins TrxQ and Sll0685) were found to be less abundant at pH 9.2 compared to pH 7.5. Moreover, the HspA chaperone was strongly reduced at pH 9.2, while the 33 kDa chaperone (Hsp33) was more abundant at pH 9.2. However, two proteins of unknown function, Sll1862 and Sll1863, that have been described as general stress proteins (Klähn et al. 2021), exhibited increased abundance at pH 9.2. Thus, there is no evident stress response under either pH condition for *Synechocystis*.

#### 3.3.4 Nitrogen Assimilation and Protein Synthesis

Inorganic nitrogen sources are converted to ammonium in the cell before amino acid biosynthesis. Nitrate imported from the medium to the cell is reduced by the nitrate reductase (NarB) and further converted to ammonium by the action of nitrite reductase (NirA). Intriguingly, NrtC, part of the nitrate transporter complex, was found upregulated at pH 9.2 while NirA diminished (Figure 4). The gene encoding NirA is putatively in the same operon as cyanase (CynS, Slr0899), which was also found less abundant. Cyanase catalyzes the reaction of cyanate (OCN^−^) with bicarbonate to produce ammonia and CO_2_ (Johnson and Anderson 1987). The glutamine synthetase (GS) type III (glnN, slr0288), which is responsible for the condensation of ammonium and glutamate to form glutamine (García‐Domínguez et al. 1997), was also found less abundant. The type I GS enzyme in *Synechocystis*, GlnA, was also slightly reduced (Log_2_FC = −0.52, *p* = 0.01). In line, IF17 (GifB, Sll1515), an inhibitory protein for GS activity, was found strongly upregulated. Glutamate synthase (GOGAT) converts glutamine and 2‐oxoglutarate to form two molecules of glutamate. *Synechocystis* has two GOGATs: the NAD(P)H‐dependent GOGAT formed by the GltB/GltD complex and the ferredoxin‐dependent GOGAT GlsF. The lower abundance of GltB and GlsF proteins indicated diminished content of both types of enzymes at pH 9.2. Consistent with the downregulation of nitrogen assimilation, ArgD, an enzyme catalyzing amination steps in the arginine biosynthesis pathway, and threonine synthase (ThrC), involved in L‐threonine biosynthesis, were both less abundant at pH 9.2. Accordingly, PirA (Ssr0692), a negative regulator of arginine synthesis (Bolay et al. 2021), was strongly enhanced. Arginine functions as a strong base at physiological pH due to its guanidine group, which readily accepts protons. Hence, specific downregulation of the arginine biosynthesis pathway may help maintain cytosolic pH homeostasis under alkaline external pH. Furthermore, 3‐deoxy‐D‐arabinoheptulosonate‐7‐phosphate (DAHP) synthase (Sll0934), which functions in the early steps of phenylalanine and tyrosine biosynthesis (Brey et al. 2020), also showed a reduced content.

At alkaline pH, some bacteria upregulate amino acid deaminases or catabolic pathways to produce organic acids (Krulwich et al. 2011; Padan et al. 2005). This observation has also been made earlier in *Synechocystis*; L‐threonine deaminase and several genes involved in the biosynthesis of the branched‐chain amino acids were upregulated in short‐term shifts (up to 6 h) from pH 7.5 to pH 10.0 (Summerfield and Sherman 2008). We did not detect any changes in abundance for amino acid deaminases; however, the alternative group 2 sigma factor, SigE, was strongly upregulated. The SigE activates the expressions of genes in sugar catabolic pathways (Osanai et al. 2011) and has a role in acclimation to salt stress (Tyystjärvi et al. 2013) and nitrogen starvation (Muro‐Pastor et al. 2001). It is worth noting that the Mg‐chelatase subunit ChlH acts as an anti‐sigma factor by binding SigE (Osanai et al. 2009) and was also slightly diminished.

Based on our results, longer‐term acclimation to high pH in *Synechocystis* does not involve enhanced amino acid biosynthesis and their catabolism into organic acids. However, the increased abundance of SigE suggests a potential increase in sugar catabolism. Intriguingly, we found a widespread downregulation of nitrogen assimilation and a specific reduction of the arginine biosynthesis pathway at higher pH. Several adjustments in metal uptake proteins might be connected to reduced protein synthesis, since up to one‐third of all bacterial proteins contain metal cofactors (Murdoch and Skaar 2022).

#### 3.3.5 Proteins Involved in CO_2_ Concentrating Mechanism

Subunits of the ATP‐dependent bicarbonate transporter BCT1‐complex, CmpB‐D, increased in abundance at pH 9.2 compared to pH 7.5 or pH 8.2, while the sodium‐dependent bicarbonate transporter SbtA showed a reduced abundance (Figures 3, 4). Accordingly, the abundance of the SbtA regulator, PII‐type protein SbtB, was also decreased. The other sodium‐dependent bicarbonate transporter in *Synechocystis*, BicA, was not detected in this study. The SbtA and BCT1‐complex are known to be strongly induced in ambient CO_2_ conditions, whereas the expression of BicA is constitutive or even increases in 3% CO_2_ (Billis et al. 2014; Eisenhut et al. 2007; Muth‐Pawlak et al. 2022). NdhR (CcmR) is a central regulator of carbon metabolism in *Synechocystis* and a repressor of *sbtA* and the *ndhF3/ndhD3/cupA/cupS* operon. In accordance, the CupS, a subunit of NDH‐1_3_ complex functioning in CO_2_ uptake, was found to be diminished at pH 9.2, as well as the NdhA, a core subunit present in all NDH‐1 complexes.

### 3.4 Contribution of Different HCO_3_ ^−^ Transporters in Cells Grown at Alkaline pH

**FIGURE 5:**
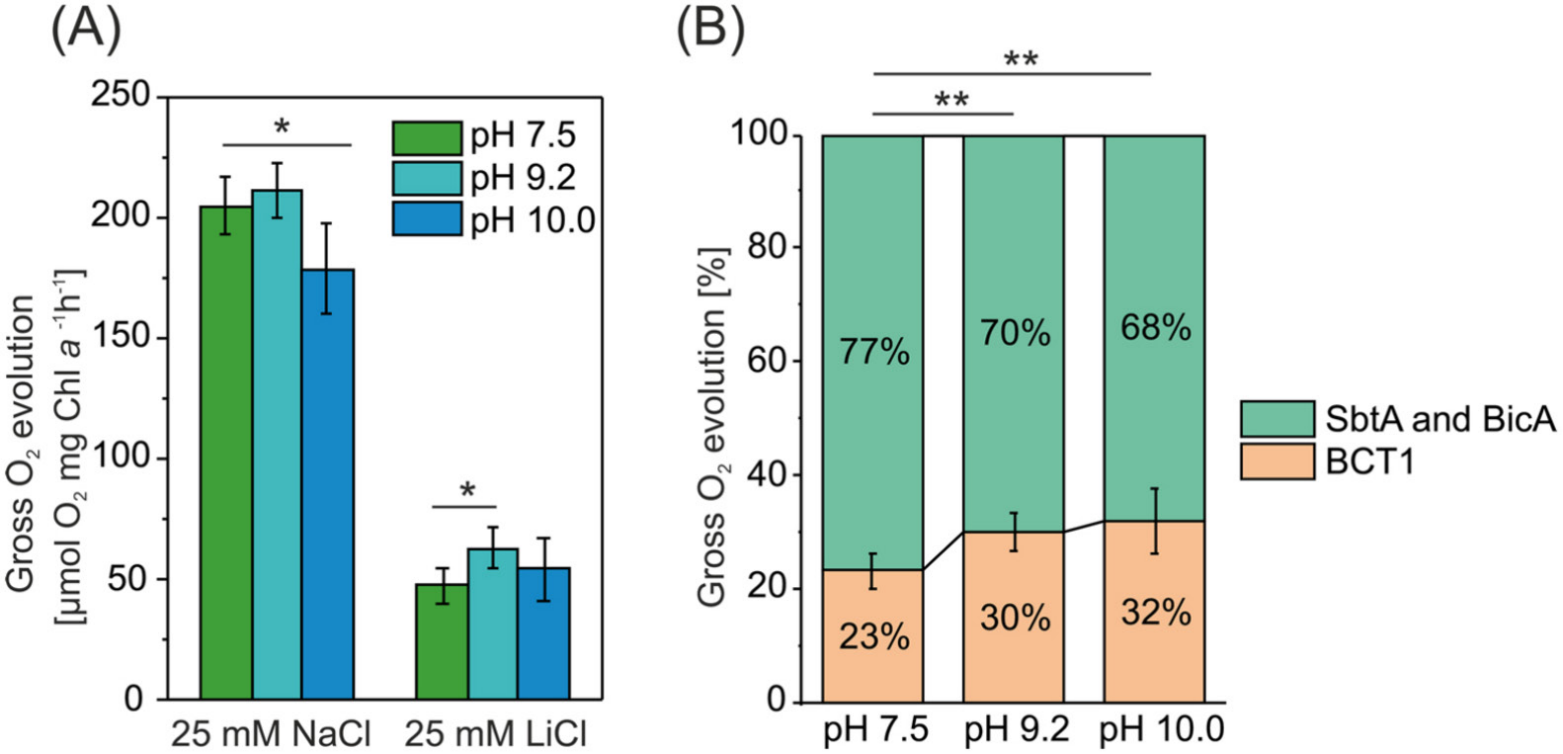
Gross O_2_ evolution of *Synechocystis* grown at different pH. (A) The rate of the gross O_2_ evolution was used for indirect assessment of HCO_3_^−^ consumption. Cells were suspended in BG‐11 medium without Na^+^ and buffered at pH 10.0. The gross O_2_ evolution rate was measured in the presence of 25 mM NaCl. To assess the activity of the BCT1 transporter complex, 25 mM LiCl was added to inhibit sodium dependent bicarbonate transporters SbtA and BicA. (B) Contribution of BCT1 activity to the gross O_2_ evolution rate, determined by the difference in oxygen evolution with and without LiCl treatment. Error bars indicate standard deviations (*n* = 5). The asterisks denote a significant difference between cells cultivated in medium with pH 7.5 and higher pH, as determined by Student's *t* test (*p < 0.05, **p < 0.01, ***p < 0.001).

The pH induced changes in HCO_3_^−^ transporter abundance revealed by proteomics analysis prompted us to evaluate the HCO_3_^−^ uptake capacity in cells grown at various pH levels. To evaluate differences in the HCO_3_^−^ uptake, we measured the photosynthetic gross oxygen evolution using the Membrane Inlet Mass Spectroscopy (MIMS) technique. The experiments were performed by monitoring O_2_ evolution in modified BG‐11 medium without Na^+^ and buffered with 20 mM CHES‐KOH pH 10.0, where practically all dissolved Ci exists as HCO_3_^−^ or as CO_3_^2−^ (Table S1). The absence of CO_2_ was confirmed by monitoring CO_2_ (m/z = 44) during gas exchange measurements, ensuring that all Ci uptake relied on HCO_3_^−^ transporters, with no contribution from CO_2_ uptake via NDH‐1_3_ and NDH‐1_4_ complexes, although these still contribute to internal Ci equilibration. We added 25 mM NaCl to enable sodium‐dependent bicarbonate uptake by SbtA and BicA. The gross O_2_ evolution rate was determined after 3.5 min upon illumination, as it is known that HCO_3_^−^ ‐dependent O_2_ evolution takes up to 3 min to reach steady rates (Price et al. 2004). The gross O_2_ evolution rate of *Synechocystis* did not differ between WT cells grown at pH 7.5 and those grown at pH 9.2 (Figure 5A). However, at pH 10.0, the oxygen evolution rate was lower compared to pH 7.5, despite the fact that cells exhibited faster growth at pH 10.0 than at the lower pH‐7.5 (Figure 1A). This may be explained by the lower Chl *a* content per cell at pH 7.5 compared to cells grown at pH 10.0 (Figure 1E), since the oxygen evolution measurements were normalized based on Chl *a* content.

To evaluate HCO_3_^−^ uptake via the BCT1 complex, we added 25 mM LiCl prior to the measurement, as lithium ions are known to inhibit sodium‐dependent carbonate transport systems (Espie et al. 1988; Jiang et al. 2013; Sandrini et al. 2015). In the presence of lithium ions, the gross O_2_ evolution rate accounted for 23% ± 2.7%, 30% ± 3.1%, and 32% ± 5.5% of the total rate in cells grown at pH 7.5, 9.2, and 10.0, respectively (Figure 5B). These results suggest an increased BCT1 activity at higher pH, in accordance with elevated BCT1 protein levels and reduced SbtA levels observed at pH 9.2 (Figure 3).

To confirm the contribution of the bicarbonate transporters in photosynthetic activity, we measured the O_2_ evolution in the Δ4 strain (Δ*ndhD3*/*ndhD4*/*sbtA*/*cmpA*; Shibata et al. 2002). This strain, which retains only the BicA transporter, was grown at pH 8.2, as it does not grow under other pH conditions at air‐level CO_2_ used in this study. As expected, the gross O_2_ evolution rate in the presence of NaCl was slightly lower than in the WT, indicating that the remaining BicA transporter was active and able to partially compensate for the loss of the other Ci sequestration systems (Figure S3). Addition of LiCl further suppressed gross O_2_ evolution by inactivating BicA, resulting in a residual O_2_ evolution most probably driven by passive CO_2_ diffusion into the cells. However, it is difficult to draw firm conclusions about the activity of specific transporters based solely on the deletion strains, as cells can partially compensate for the loss of other transporters. In agreement with this, *bicA* expression was found to be constitutively upregulated in the Δ4 strain and the carboxysome content was increased compared to the WT (Orf et al. 2015).

There were two main reasons why we did not evaluate the contribution of individual transporters by measuring CO_2_ exchange with MIMS at the pH range (7.5–10.0) used in this study: (1) when cells are adjusted to pH > 9, the CO_2_ concentration in the medium is negligible and falls below the measuring detection threshold of the MIMS method even upon addition of NaHCO_3_; (2) In pH < 10, it is not possible to exclude the activity of NDH‐1 complexes, as indicated by the gross O_2_ evolution rate, which showed no difference in the presence or absence of LiCl in cells adjusted to pH 7.5 (Figure S4A).

At pH 9.2 and 10.0, total Ci and bicarbonate concentrations are higher compared to pH 7.5 (Table S1; Price 2011). Therefore, the cells can maintain faster growth at least up to pH 10.0, as tested in this study (Figure 1A). Even though total dissolved Ci is increasing with increasing pH, the BCT1 subunits were more abundant at high external pH and higher amount of the BCT1 complex was reflected on the BCT1 activity (Figures 2 and 5). Previously, it has been observed on transcript level that the cells upregulate both *sbtA* and genes from the *cmp*‐operon at pH 10.0 in comparison to pH 7.5 (Summerfield and Sherman 2008). Both SbtA and BCT1 components are additionally posttranslationally regulated by multiple phosphorylations (Angeleri et al. 2016; Spät et al. 2021). CmpA, the periplasmic bicarbonate binding protein, and CmpB, the membrane integrated permease subunit of BCT1, both showed significant changes in phosphorylation events upon the shift from 5% CO_2_ to ambient CO_2_ (Spät et al. 2021). The activity of HCO_3_^−^ uptake in *Synechocystis* has been shown to be induced within minutes, and this rapid induction can be inhibited by common protein kinase inhibitors, suggesting that it is mediated by phosphorylation events (Amoroso et al. 2003). The response of the phosphorylation levels of bicarbonate transporters to changes in external pH is not known, but might play a role at least in rapidly changing pH environments.

It is also interesting that the ΔpH between the lumen and cytosol increases with increasing environmental pH, thus increasing ATP synthase activity (Belkin et al. 1987; Morris et al. 2016). Because BCT1 is ATP‐dependent, it is plausible that the BCT1 abundance is increased upon higher ATP levels, while the SbtA amount and activity are proportionally lower than in neutral pH. Moreover, given that the BCT1 complex is primarily found in freshwater cyanobacteria and it is absent in most marine strains (Klanchui et al. 2017), it is possible that Na^+^‐dependent bicarbonate transport becomes less favorable in freshwater cyanobacteria at pH > 9 due to limited Na^+^ availability for maintaining the ion gradient across the membrane. Both BicA and SbtA transporters require at least 1 mM Na^+^ concentration to function (Shibata et al. 2002), whereas Na^+^ concentration in freshwaters may often remain below this threshold.

To evaluate the direct impact of LiCl on photosynthetic electron transport, gross O_2_ evolution was measured by MIMS, alongside Chl *a* fluorescence induction and flash‐induced fluorescence yield and its subsequent relaxation in darkness (Figure S4). These measurements were conducted in *Synechocystis* cells grown at pH 7.5, a condition where dissolved CO_2_ is expected to fully compensate for Ci import. Therefore, no changes were expected when Na^+^‐dependent bicarbonate transporters were inhibited by LiCl. Indeed, no significant differences were observed between control and LiCl‐treated cells grown at pH 7.5 in any of the measured photosynthetic parameters (Figure S4), indicating that short‐term exposure to LiCl does not directly affect photosynthetic electron transport. The significant reduction in gross O_2_ evolution rates upon LiCl addition at pH 10.0 is attributed to the inactivation of bicarbonate transporters, rather than alterations in PSII performance.

## 4 Conclusions

This study revealed that while increasing the external growth medium from pH 7.5 to 8.2 induced only minor proteomic changes, primarily in plasma membrane‐associated proteins, growth at pH 9.2 resulted in widespread differences in protein abundances in *Synechocystis*. Specifically, many transporters and regulatory proteins showed altered abundance compared to cells grown at pH 7.5 or 8.2. Intriguingly, the cation/H^+^ antiporters, which play a crucial role in pH homeostasis, remained largely unchanged. This suggests that their constitutive expression enables a rapid response to external pH fluctuations, with potential posttranslational regulation of their activity. Furthermore, the observed modifications in the composition of bicarbonate transporters, with an increased abundance and activity of ATP‐dependent BCT1‐complex at pH 9.2, alongside a decrease in the Na‐dependent bicarbonate transporter SbtA, may indicate a cyanobacteria‐specific acclimation strategy to alkaline conditions. Investigating whether the Na‐dependent bicarbonate transporters exhibit pH‐dependent expression in other cyanobacterial species could provide further insights into this adaptive mechanism. In addition, our observation of changes in pigment composition per cell at different pH conditions suggests profound physiological adjustments at divergent pH levels. A deeper understanding of cyanobacterial acclimation to high pH has practical implications for applied research, as optimizing growth conditions and cultivation media is essential for many biotechnological applications. Furthermore, the natural preference of cyanobacteria for alkaline environments may offer a selective advantage in large‐scale cultivation, as high pH can suppress the growth of heterotrophic contaminants that typically thrive at neutral pH.

## Author Contributions

Y.A. conceived the study. H.M., M.H., D.M.‐P., and Y.A. designed the research. H.M., M.H., and D.M.‐P. performed the experiments. H.M. analyzed the data and prepared the first draft of the manuscript. H.M. and Y.A. finalized the manuscript with contributions from M.H. All authors approved the final version of the manuscript.

## Supporting information


**Figure S1:** Total protein concentration in a cell lysate.
**Figure S2:** Properties of *Synechocystis*.
**Figure S3:** Gross O_2_ evolution of WT and Δ4 strain.
**Figure S4:** Characterization of PSII in the presence of LiCl in cells cultured and measured at pH 7.5.
**Table S1:** Predicted pH and inorganic carbon species in cell‐free BG‐11 medium adjusted to different pH.
**Table S2:** The pH of the BG‐11 used for the cultivations and the pH of the culture supernatant.
**Table S3:** Cell size of *Synechocystis*.

**Table S4:** Proteins identified in pH 7.5, pH 8.2, or pH 9.2 condition.
**Table S5:** Proteins quantified in pH 8.2 and pH 9.2 conditions in comparison to pH 7.5. The statistically significant results (*p* < 0.05) with fold change threshold (−0.58 > Log2FC > 0.58) are highlighted with bold font.
**Table S6:** Proteome data used for Figure 3.

## Data Availability

The mass spectrometry proteomics data that support the findings of this study are available in ProteomeXchange Consortium via the PRIDE (http://www.ebi.ac.uk/pride) partner repository with dataset identifier PXD061233 and project https://doi.org/10.6019/PXD061233.

## References

[ppl70474-bib-0001] Amoroso, G. , N. Seimetz , and D. Sültemeyer . 2003. “The dc13 Gene Upstream of ictB Is Involved in Rapid Induction of the High Affinity Na^+^ Dependent HCO_3_ ^−^ Transporter in Cyanobacteria.” Photosynthesis Research 77, no. 2–3: 127–138. 10.1023/A:1025873718682. 16228371

[ppl70474-bib-0002] Angeleri, M. , D. Muth‐Pawlak , E. M. Aro , and N. Battchikova . 2016. “Study of O‐Phosphorylation Sites in Proteins Involved in Photosynthesis‐Related Processes in *Synechocystis* sp. Strain PCC 6803: Application of the SRM Approach.” Journal of Proteome Research 15, no. 12: 4638–4652. 10.1021/acs.jproteome.6b00732. 27790906

[ppl70474-bib-0003] Assayag, N. , K. Rivé , M. Ader , D. Jézéquel , and P. Agrinier . 2006. “Improved Method for Isotopic and Quantitative Analysis of Dissolved Inorganic Carbon in Natural Water Samples.” Rapid Communications in Mass Spectrometry 20, no. 15: 2243–2251. 10.1002/RCM.2585. 16810706

[ppl70474-bib-0004] Badarau, A. , and C. Dennison . 2011. “Thermodynamics of Copper and Zinc Distribution in the Cyanobacterium *Synechocystis* PCC 6803.” Proceedings of the National Academy of Sciences of the United States of America 108, no. 32: 13007–13012. 10.1073/PNAS.1101448108. 21778408 PMC3156197

[ppl70474-bib-0005] Beckmann, K. , J. Messinger , M. R. Badger , T. Wydrzynski , and W. Hillier . 2009. “On‐Line Mass Spectrometry: Membrane Inlet Sampling.” Photosynthesis Research 102, no. 2: 511–522. 10.1007/s11120-009-9474-7. 19653116 PMC2847165

[ppl70474-bib-0006] Behera, S. , Z. Xu , L. Luoni , et al. 2018. “Cellular Ca^2+^ Signals Generate Defined pH Signatures in Plants.” Plant Cell 30, no. 11: 2704–2719. 10.1105/tpc.18.00655. 30377237 PMC6305977

[ppl70474-bib-0007] Belkin, S. , R. J. Mehlhorn , and L. Packer . 1987. “Proton Gradients in Intact Cyanobacteria.” Plant Physiology 84: 25–30. 11539679 10.1104/pp.84.1.25 PMC1056521

[ppl70474-bib-0008] Bernal, M. , D. Casero , V. Singh , et al. 2012. “Transcriptome Sequencing Identifies SPL7‐Regulated Copper Acquisition Genes FRO4/FRO5 and the Copper Dependence of Iron Homeostasis in Arabidopsis.” Plant Cell 24, no. 2: 738–761. 10.1105/tpc.111.090431. 22374396 PMC3315244

[ppl70474-bib-0009] Billini, M. , K. Stamatakis , and V. Sophianopoulou . 2008. “Two Members of a Network of Putative Na+/H+ Antiporters Are Involved in Salt and pH Tolerance of the Freshwater Cyanobacterium *Synechococcus elongatus* .” Journal of Bacteriology 190, no. 19: 6318–6329. 10.1128/JB.00696-08. 18641132 PMC2565994

[ppl70474-bib-0010] Billis, K. , M. Billini , H. J. Tripp , N. C. Kyrpides , and K. Mavromatis . 2014. “Comparative Transcriptomics Between Synechococcus PCC 7942 and *Synechocystis* PCC 6803 Provide Insights Into Mechanisms of Stress Acclimation.” PLoS One 9, no. 10: 1–10. 10.1371/journal.pone.0109738. PMC4207680 25340743

[ppl70474-bib-0011] Blanco‐Rivero, A. , F. Leganés , E. Fernández‐Valiente , P. Calle , and F. Fernández‐Piñas . 2005. “mrpA, a Gene With Roles in Resistance to Na+ and Adaptation to Alkaline pH in the Cyanobacterium *Anabaena* sp. PCC7120.” Microbiology 151, no. 5: 1671–1682. 10.1099/mic.0.27848-0. 15870474

[ppl70474-bib-0012] Bolay, P. , R. Rozbeh , M. I. Muro‐Pastor , et al. 2021. “The Novel P II‐Interacting Protein PirA Controls Flux Into the Cyanobacterial Ornithine‐Ammonia Cycle.” 10.1128/mBio. PMC8092223 33758091

[ppl70474-bib-0013] Brey, L. F. , A. J. Włodarczyk , J. F. Bang Thøfner , et al. 2020. “Metabolic Engineering of *Synechocystis* sp. PCC 6803 for the Production of Aromatic Amino Acids and Derived Phenylpropanoids.” Metabolic Engineering 57: 129–139. 10.1016/j.ymben.2019.11.002. 31722246

[ppl70474-bib-0014] Carrasquer‐Alvarez, E. , U. A. Hoffmann , A. S. Geissler , et al. 2025. “Photosynthesis in *Synechocystis* sp. PCC 6803 Is Not Optimally Regulated Under Very High CO_2_ .” Applied Microbiology and Biotechnology 109, no. 1: 1–19. 10.1007/S00253-025-13416-2. 39883173 PMC11782454

[ppl70474-bib-0015] Chaves, J. E. , H. Kirst , and A. Melis . 2015. “Isoprene Production in *Synechocystis* Under Alkaline and Saline Growth Conditions.” Journal of Applied Phycology 27, no. 3: 1089–1097. 10.1007/s10811-014-0395-2.

[ppl70474-bib-0016] Cole, J. J. , and Y. T. Prairie . 2014. “Dissolved CO_2_ in Freshwater Systems.” In Reference Module in Earth Systems and Environmental Sciences. Elsevier. 10.1016/B978-0-12-409548-9.09399-4.

[ppl70474-bib-0017] Coleman, J. R. , and B. Colman . 1981. “Inorganic Carbon Accumulation and Photosynthesis in a Blue‐Green Alga as a Function of External pH.” Plant Physiology 67: 917–921. 16661792 10.1104/pp.67.5.917 PMC425800

[ppl70474-bib-0018] Eisenhut, M. , E. A. Von Wobeser , L. Jonas , et al. 2007. “Long‐Term Response Toward Inorganic Carbon Limitation in Wild Type and Glycolate Turnover Mutants of the Cyanobacterium *Synechocystis* sp. Strain PCC 6803.” Plant Physiology 144, no. 4: 1946–1959. 10.1104/pp.107.103341. 17600135 PMC1949882

[ppl70474-bib-0019] Elanskaya, I. V. , I. V. Karandashova , A. V. Bogachev , and M. Hagemann . 2002. “Functional Analysis of the Na+/H+ Antiporter Encoding Genes of the Cyanobacterium *Synechocystis* PCC 6803.” Biochemistry. Biokhimiia 67, no. 4: 432–440. 10.1023/A:1015281906254. 11996656

[ppl70474-bib-0020] Espie, G. S. , A. G. Miller , and D. T. Canvin . 1988. “Characterization of the Na+‐Requirement in Cyanobacterial Photosynthesis'.” Plant Physiology 88: 757–763. 16666379 10.1104/pp.88.3.757 PMC1055656

[ppl70474-bib-0021] Fulda, S. , F. Huang , F. Nilsson , M. Hagemann , and B. Norling . 2000. “Proteomics of *Synechocystis* sp. Strain PCC 6803: Identification of Periplasmic Proteins in Cells Grown at Low and High Salt Concentrations.” European Journal of Biochemistry 267, no. 19: 5900–5907. 10.1046/j.1432-1327.2000.01642.x. 10998049

[ppl70474-bib-0022] García‐Cañas, R. , J. Giner‐Lamia , F. J. Florencio , and L. López‐Maury . 2021. “A Protease‐Mediated Mechanism Regulates the Cytochrome *c* _6_/Plastocyanin Switch in *Synechocystis* sp. PCC 6803.” Proceedings of the National Academy of Sciences 118, no. 5: e2017898118. 10.1073/pnas.2017898118. PMC7865156 33495331

[ppl70474-bib-0023] García‐Domínguez, M. , J. C. Reyes , and F. J. Florencio . 1997. “Purification and Characterization of a New Type of Glutamine Synthetase From Cyanobacteria.” European Journal of Biochemistry 244, no. 1: 258–264. 10.1111/j.1432-1033.1997.00258.x. 9063472

[ppl70474-bib-0024] Giner‐Lamia, J. , L. López‐Maury , J. C. Reyes , and F. J. Florencio . 2012. “The CopRS Two‐Component System Is Responsible for Resistance to Copper in the Cyanobacterium *Synechocystis* sp. PCC 6803.” Plant Physiology 159, no. 4: 1806–1818. 10.1104/pp.112.200659. 22715108 PMC3425214

[ppl70474-bib-0025] Heberle, H. , V. G. Meirelles , F. R. da Silva , G. P. Telles , and R. Minghim . 2015. “InteractiVenn: A Web‐Based Tool for the Analysis of Sets Through Venn Diagrams.” BMC Bioinformatics 16, no. 1: 169. 10.1186/s12859-015-0611-3. 25994840 PMC4455604

[ppl70474-bib-0026] Hihara, Y. , K. Sonoike , M. Kanehisa , and M. Ikeuchi . 2003. “DNA Microarray Analysis of Redox‐Responsive Genes in the Genome of the Cyanobacterium *Synechocystis* sp. Strain PCC 6803.” Journal of Bacteriology 185, no. 5: 1719–1725. 10.1128/JB.185.5.1719-1725.2003. 12591891 PMC148065

[ppl70474-bib-0027] Hirose, Y. , T. Shimada , R. Narikawa , M. Katayama , and M. Ikeuchi . 2008. “Cyanobacteriochrome CcaS Is the Green Light Receptor That Induces the Expression of Phycobilisome Linker Protein.” Proceedings of the National Academy of Sciences of the United States of America 105: 9528–9533. 10.1073/pnas.0801826105. 18621684 PMC2474522

[ppl70474-bib-0028] Ito, M. , M. Morino , and T. A. Krulwich . 2017. “Mrp Antiporters Have Important Roles in Diverse Bacteria and Archaea.” Frontiers in Microbiology 8: 2325. 10.3389/fmicb.2017.02325. 29218041 PMC5703873

[ppl70474-bib-0029] Jahn, M. , V. Vialas , J. Karlsen , et al. 2018. “Growth of Cyanobacteria Is Constrained by the Abundance of Light and Carbon Assimilation Proteins.” Cell Reports 25, no. 2: 4827. 10.1016/j.celrep.2018.09.040. 30304686

[ppl70474-bib-0030] Jiang, H. B. , H. M. Cheng , K. S. Gao , and B. S. Qiu . 2013. “Inactivation of Ca^2+^/H^+^ Exchanger in *Synechocystis* sp. Strain PCC 6803 Promotes Cyanobacterial Calcification by Upregulating CO_2_‐Concentrating Mechanisms.” Applied and Environmental Microbiology 79, no. 13: 4048–4055. 10.1128/AEM.00681-13. 23624472 PMC3697565

[ppl70474-bib-0031] Johnson, W. V. , and P. M. Anderson . 1987. “Bicarbonate Is a Recycling Substrate for Cyanase.” Journal of Biological Chemistry 262, no. 19: 9021–9025. 10.1016/S0021-9258(18)48040-4. 3110153

[ppl70474-bib-0032] Käll, L. , J. D. Canterbury , J. Weston , W. S. Noble , and M. J. MacCoss . 2007. “Semi‐Supervised Learning for Peptide Identification From Shotgun Proteomics Datasets.” Nature Methods 4, no. 11: 923–925. 10.1038/nmeth1113. 17952086

[ppl70474-bib-0033] Kaneko, T. , S. Sato , H. Kotani , et al. 1996. “Sequence Analysis of the Genome of the Unicellular Cyanobacterium *Synechocystis* sp. Strain PCC6803. II. Sequence Determination of the Entire Genome and Assignment of Potential Protein‐Coding Regions (Supplement).” DNA Research 3, no. 3: 109–136. 10.1093/dnares/3.3.185. 8905231

[ppl70474-bib-0034] Klähn, S. , S. Mikkat , M. Riediger , J. Georg , W. R. Hess , and M. Hagemann . 2021. “Integrative Analysis of the Salt Stress Response in Cyanobacteria.” Biology Direct 16, no. 1: 26. 10.1186/s13062-021-00316-4. 34906211 PMC8670252

[ppl70474-bib-0035] Klanchui, A. , S. Cheevadhanarak , P. Prommeenate , and A. Meechai . 2017. “Exploring Components of the CO_2_‐Concentrating Mechanism in Alkaliphilic Cyanobacteria Through Genome‐Based Analysis.” Computational and Structural Biotechnology Journal 15: 340–350. 10.1016/j.csbj.2017.05.001. 28652895 PMC5472144

[ppl70474-bib-0036] Kondo, K. , Y. Ochiai , M. Katayama , and M. Ikeuchi . 2007. “The Membrane‐Associated CpcG2‐Phycobilisome in *Synechocystis*: A New Photosystem I Antenna.” Plant Physiology 144, no. 2: 1200–1210. 10.1104/pp.107.099267. 17468217 PMC1914160

[ppl70474-bib-0037] Krulwich, T. A. , G. Sachs , and E. Padan . 2011. “Molecular Aspects of Bacterial pH Sensing and Homeostasis.” Nature Reviews Microbiology 9, no. 5: 330–343. 10.1038/nrmicro2549. 21464825 PMC3247762

[ppl70474-bib-0038] Kubota, H. , I. Sakurai , K. Katayama , et al. 2010. “Purification and Characterization of Photosystem I Complex From *Synechocystis* sp. PCC 6803 by Expressing Histidine‐Tagged Subunits.” Biochimica et Biophysica Acta 1797, no. 1: 98–105. 10.1016/j.bbabio.2009.09.001. 19751700

[ppl70474-bib-0039] Kujat, S. L. , and G. W. Owttrim . 2000. “Redox‐Regulated RNA Helicase Expression.” Plant Physiology 124, no. 2: 703–713. 10.1104/PP.124.2.703. 11027719 PMC59175

[ppl70474-bib-0040] Kupriyanova, E. V. , and O. S. Samylina . 2015. “CO_2_‐Concentrating Mechanism and Its Traits in Haloalkaliphilic Cyanobacteria.” Microbiology 84, no. 2: 112–124. 10.1134/S0026261715010075. 26263620

[ppl70474-bib-0041] Kurian, D. , K. Phadwal , and P. Mäenpää . 2006. “Proteomic Characterization of Acid Stress Response in *Synechocystis* sp. PCC 6803.” Proteomics 6, no. 12: 3614–3624. 10.1002/pmic.200600033. 16691555

[ppl70474-bib-0042] López‐Archilla, A. I. , D. Moreira , P. López‐García , and C. Guerrero . 2004. “Phytoplankton Diversity and Cyanobacterial Dominance in a Hypereutrophic Shallow Lake With Biologically Produced Alkaline pH.” Extremophiles 8, no. 2: 109–115. 10.1007/s00792-003-0369-9. 15064977

[ppl70474-bib-0043] López‐Maury, L. , J. Giner‐Lamia , and F. J. Florencio . 2012. “Redox Control of Copper Homeostasis in Cyanobacteria.” Plant Signaling & Behavior 7, no. 12: 1712–1714. 10.4161/psb.22323. 23073008 PMC3578916

[ppl70474-bib-0044] Mangan, N. M. , A. Flamholz , R. D. Hood , R. Milo , and D. F. Savage . 2016. “pH Determines the Energetic Efficiency of the Cyanobacterial CO_2_ Concentrating Mechanism.” Proceedings of the National Academy of Sciences of the United States of America 113, no. 36: E5354–E5362. 10.1073/pnas.1525145113. 27551079 PMC5018799

[ppl70474-bib-0045] Marbouty, M. , K. Mazouni , C. Saguez , C. Cassier‐Chauvat , and F. Chauvat . 2009. “Characterization of the *Synechocystis* Strain PCC 6803 Penicillin‐Binding Proteins and Cytokinetic Proteins FtsQ and FtsW and Their Network of Interactions With ZipN.” Journal of Bacteriology 191, no. 16: 5123–5133. 10.1128/JB.00620-09. 19542290 PMC2725598

[ppl70474-bib-0046] Meeks, J. C. , and R. W. Castenholz . 1971. “Growth and Photosynthesis in an Extreme Thermophile, *Synechococcus lividus* (Cyanophyta).” Archiv für Mikrobiologie 78, no. 1: 25–41. 10.1007/BF00409086. 4999393

[ppl70474-bib-0047] Morris, J. N. , J. J. Eaton‐Rye , and T. C. Summerfield . 2016. “Environmental pH and the Requirement for the Extrinsic Proteins of Photosystem II in the Function of Cyanobacterial Photosynthesis.” Frontiers in Plant Science 7, no. 1: 1135. 10.3389/fpls.2016.01135. 27555848 PMC4977308

[ppl70474-bib-0048] Murdoch, C. C. , and E. P. Skaar . 2022. “Nutritional Immunity: The Battle for Nutrient Metals at the Host–Pathogen Interface.” Nature Reviews Microbiology 20, no. 11: 657–670. 10.1038/s41579-022-00745-6. 35641670 PMC9153222

[ppl70474-bib-0049] Muro‐Pastor, A. M. , A. Herrero , and E. Flores . 2001. “Nitrogen‐Regulated Group 2 Sigma Factor From *Synechocystis* sp. Strain PCC 6803 Involved in Survival Under Nitrogen Stress.” Journal of Bacteriology 183, no. 3: 1090–1095. 10.1128/JB.183.3.1090-1095.2001. 11208809 PMC94978

[ppl70474-bib-0050] Mustila, H. , D. Muth‐Pawlak , E. M. Aro , and Y. Allahverdiyeva . 2021. “Global Proteomic Response of Unicellular Cyanobacterium *Synechocystis* sp. PCC 6803 to Fluctuating Light Upon CO_2_ Step‐Down.” Physiologia Plantarum 173, no. 1: 305–320. 10.1111/ppl.13482. 34145600

[ppl70474-bib-0051] Mustila, H. , P. Paananen , N. Battchikova , et al. 2016. “The Flavodiiron Protein Flv3 Functions as a Homo‐Oligomer During Stress Acclimation and Is Distinct From the Flv1/Flv3 Hetero‐Oligomer Specific to the O_2_ Photoreduction Pathway.” Plant and Cell Physiology 57, no. 7: 1468–1483. 10.1093/pcp/pcw047. 26936793 PMC4937785

[ppl70474-bib-0052] Muth‐Pawlak, D. , S. Kreula , P. J. Gollan , T. Huokko , Y. Allahverdiyeva , and E. M. Aro . 2022. “Patterning of the Autotrophic, Mixotrophic, and Heterotrophic Proteomes of Oxygen‐Evolving Cyanobacterium *Synechocystis* sp. PCC 6803.” Frontiers in Microbiology 13: 891895. 10.3389/fmicb.2022.891895. 35694301 PMC9175036

[ppl70474-bib-0053] Nagarajan, S. , S. Srivastava , and L. A. Sherman . 2014. “Essential Role of the Plasmid hik31 Operon in Regulating Central Metabolism in the Dark in *Synechocystis* sp. PCC 6803.” Molecular Microbiology 91, no. 1: 79–97. 10.1111/mmi.12442. 24237382

[ppl70474-bib-0054] Nanatani, K. , T. Shijuku , Y. Takano , et al. 2015. “Comparative Analysis of Kdp and Ktr Mutants Reveals Distinct Roles of the Potassium Transporters in the Model Cyanobacterium *Synechocystis* sp. Strain PCC 6803.” Journal of Bacteriology 197, no. 4: 676–687. 10.1128/JB.02276-14. 25313394 PMC4334184

[ppl70474-bib-0055] Nguyen, B. T. , and B. E. Rittmann . 2016. “Effects of Inorganic Carbon and pH on Growth Kinetics of *Synechocystis* sp. PCC 6803.” Algal Research 19: 363–369. 10.1016/j.algal.2016.03.011.

[ppl70474-bib-0056] O'Hara, G. W. , T. J. Goss , M. J. Dilworth , and A. R. Glenn . 1989. “Maintenance of Intracellular pH and Acid Tolerance in *Rhizobium meliloti* .” Applied and Environmental Microbiology 55, no. 8: 1870–1876. 10.1128/AEM.55.8.1870-1876.1989. 16347984 PMC202972

[ppl70474-bib-0057] Orf, I. , S. Klähn , D. Schwarz , et al. 2015. “Integrated Analysis of Engineered Carbon Limitation in a Quadruple CO_2_/HCO_3_ ^−^ Uptake Mutant of *Synechocystis* sp. PCC 6803.” Plant Physiology 169, no. 3: 1787–1806. 10.1104/pp.15.01289. 26373660 PMC4634100

[ppl70474-bib-0058] Osanai, T. , M. Imashimizu , A. Seki , et al. 2009. “ChlH, the H Subunit of the Mg‐Chelatase, Is an Anti‐Sigma Factor for SigE in *Synechocystis* sp. PCC 6803.” Proceedings of the National Academy of Sciences of the United States of America 106, no. 16: 6860–6865. 10.1073/PNAS.0810040106. 19342483 PMC2672518

[ppl70474-bib-0059] Osanai, T. , A. Oikawa , M. Azuma , et al. 2011. “Genetic Engineering of Group 2 σ Factor SigE Widely Activates Expressions of Sugar Catabolic Genes in *Synechocystis* Species PCC 6803.” Journal of Biological Chemistry 286, no. 35: 30962–30971. 10.1074/jbc.M111.231183. 21757761 PMC3162455

[ppl70474-bib-0060] Padan, E. , E. Bibi , M. Ito , and T. A. Krulwich . 2005. “Alkaline pH Homeostasis in Bacteria: New Insights.” Biochimica et Biophysica Acta 1717, no. 2: 67–88. 10.1016/j.bbamem.2005.09.010. 16277975 PMC3072713

[ppl70474-bib-0061] Paynter, J. J. , I. Andres‐Enguix , P. W. Fowler , et al. 2010. “Functional Complementation and Genetic Deletion Studies of KirBac Channels: Activatory Mutations Highlight Gating‐Sensitive Domains.” Journal of Biological Chemistry 285, no. 52: 40754–40761. 10.1074/jbc.M110.175687. 20876570 PMC3003375

[ppl70474-bib-0062] Perez‐Riverol, Y. , C. Bandla , D. J. Kundu , et al. 2025. “The PRIDE Database at 20 Years: 2025 Update.” Nucleic Acids Research 53, no. D1: D543–D553. 10.1093/NAR/GKAE1011. 39494541 PMC11701690

[ppl70474-bib-0063] Perkins, D. N. , D. J. Pappin , D. M. Creasy , and J. S. Cottrell . 1999. “Probability‐Based Protein Identification by Searching Sequence Databases Using Mass Spectrometry Data.” Electrophoresis 20, no. 18: 3551–3567. 10.1002/(SICI)1522-2683(19991201)20:18<3551::AID-ELPS3551>3.0.CO;2-2. 10612281

[ppl70474-bib-0064] Price, G. D. 2011. “Inorganic Carbon Transporters of the Cyanobacterial CO2 Concentrating Mechanism.” Photosynthesis Research 109, no. 1–3: 47–57. 10.1007/s11120-010-9608-y. 21359551

[ppl70474-bib-0065] Price, G. D. , M. R. Badger , F. J. Woodger , and B. M. Long . 2008. “Advances in Understanding the Cyanobacterial CO_2_‐Concentrating‐Mechanism (CCM): Functional Components, Ci Transporters, Diversity, Genetic Regulation and Prospects for Engineering Into Plants.” Journal of Experimental Botany 59, no. 7: 1441–1461. 10.1093/JXB/ERM112. 17578868

[ppl70474-bib-0066] Price, G. D. , F. J. Woodger , M. R. Badger , S. M. Howitt , and L. Tucker . 2004. “Identification of a SulP‐Type Bicarbonate Transporter in Marine Cyanobacteria.” Proceedings of the National Academy of Sciences of the United States of America 101, no. 52: 18228–18233. 10.1073/PNAS.0405211101. 15596724 PMC539743

[ppl70474-bib-0067] Reis, M. , S. Zenker , P. Viehöver , K. Niehaus , A. Bräutigam , and M. Eisenhut . 2024. “Study of Excess Manganese Stress Response Highlights the Central Role of Manganese Exporter Mnx for Holding Manganese Homeostasis in the Cyanobacterium *Synechocystis* sp. PCC 6803.” Microbiology 170, no. 11: 001515. 10.1099/MIC.0.001515. 39508727 PMC11649195

[ppl70474-bib-0068] Ritchie, R. J. 1991. “Membrane Potential and pH Control in the Cyanobacterium Synechococcus R‐2 ( *Anacystis nidulans* ) PCC 7942.” Journal of Plant Physiology 137, no. 4: 409–418. 10.1016/S0176-1617(11)80309-3.

[ppl70474-bib-0069] Ritter, S. P. A. , A. C. Lewis , S. L. Vincent , et al. 2020. “Evidence for Convergent Sensing of Multiple Abiotic Stresses in Cyanobacteria.” Biochimica et Biophysica Acta – General Subjects 1864, no. 1: 129462. 10.1016/j.bbagen.2019.129462. 31669584

[ppl70474-bib-0070] Sandrini, G. , S. Cunsolo , J. M. Schuurmans , H. C. P. Matthijs , and J. Huisman . 2015. “Changes in Gene Expression, Cell Physiology and Toxicity of the Harmful Cyanobacterium *Microcystis aeruginosa* at Elevated CO_2_ .” Frontiers in Microbiology 6, no. 1: 401. 10.3389/fmicb.2015.00401. 25999931 PMC4419860

[ppl70474-bib-0071] Santana‐Sanchez, A. , D. Solymosi , H. Mustila , L. Bersanini , E.‐M. Aro , and Y. Allahverdiyeva . 2019. “Flavodiiron Proteins 1‐To‐4 Function in Versatile Combinations in O_2_ Photoreduction in Cyanobacteria.” eLife 8: e45766. 10.7554/eLife.45766. 31294693 PMC6658166

[ppl70474-bib-0072] Satoh, K. , and Y. Yamamoto . 2007. “The Carboxyl‐Terminal Processing of Precursor D1 Protein of the Photosystem II Reaction Center.” Photosynthesis Research 94, no. 2–3: 203–215. 10.1007/S11120-007-9191-Z. 17551844

[ppl70474-bib-0073] Shibata, M. , H. Katoh , M. Sonoda , et al. 2002. “Genes Essential to Sodium‐Dependent Bicarbonate Transport in Cyanobacteria: Function and Phylogenetic Analysis.” Journal of Biological Chemistry 277, no. 21: 18658–18664. 10.1074/jbc.M112468200. 11904298

[ppl70474-bib-0074] Singh, S. , I. S. Thakur , and A. Singh . 1996. “Regulation of pH Homeostasis in the Cyanobacterium Hapalosiphon Hybernicus West.” Journal of General and Applied Microbiology 42, no. 1: 71–79. 10.2323/JGAM.42.71.

[ppl70474-bib-0075] Spät, P. , T. Barske , B. Maček , and M. Hagemann . 2021. “Alterations in the CO_2_ Availability Induce Alterations in the Phosphoproteome of the Cyanobacterium *Synechocystis* sp. PCC 6803.” New Phytologist 231: 1123–1137. 10.1111/nph.17423. 34058021

[ppl70474-bib-0076] Summerfield, T. C. , T. S. Crawford , R. D. Young , et al. 2013. “Environmental pH Affects Photoautotrophic Growth of *Synechocystis* sp. PCC 6803 Strains Carrying Mutations in the Lumenal Proteins of PSII.” Plant and Cell Physiology 54, no. 6: 859–874. 10.1093/pcp/pct036. 23444302

[ppl70474-bib-0077] Summerfield, T. C. , and L. A. Sherman . 2008. “Global Transcriptional Response of the Alkali‐Tolerant Cyanobacterium *Synechocystis* sp. Strain PCC 6803 to a pH 10 Environment.” Applied and Environmental Microbiology 74, no. 17: 5276–5284. 10.1128/AEM.00883-08. 18606800 PMC2546634

[ppl70474-bib-0078] Taglicht, D. , E. Padan , and S. Schuldiner . 1991. “Overproduction and Purification of a Functional Na+/H+ Antiporter Coded by nhaA (Ant) From *Escherichia coli* .” Journal of Biological Chemistry 266, no. 17: 11289–11294. 10.1016/S0021-9258(18)99161-1. 1645730

[ppl70474-bib-0079] Touloupakis, E. , B. Cicchi , A. M. S. Benavides , and G. Torzillo . 2016. “Effect of High pH on Growth of *Synechocystis* sp. PCC 6803 Cultures and Their Contamination by Golden Algae (*Poterioochromonas* sp.).” Applied Microbiology and Biotechnology 100, no. 3: 1333–1341. 10.1007/S00253-015-7024-0. 26541331 PMC4717179

[ppl70474-bib-0080] Tsujii, M. , A. Kobayashi , A. Kano , et al. 2025. “Na^+^−Driven pH Regulation by Na^+^/H^+^ Antiporters Promotes Photosynthetic Efficiency in Cyanobacteria.” Plant Physiology 197, no. 1: 562. 10.1093/PLPHYS/KIAE562. PMC11663708 39446395

[ppl70474-bib-0081] Tyystjärvi, T. , T. Huokko , S. Rantamäki , and E. Tyystjärvi . 2013. “Impact of Different Group 2 Sigma Factors on Light Use Efficiency and High Salt Stress in the Cyanobacterium *Synechocystis* sp. PCC 6803.” PLoS One 8, no. 4: e63020. 10.1371/journal.pone.0063020. 23638176 PMC3637157

[ppl70474-bib-0082] Waldron, K. J. , S. Tottey , S. Yanagisawa , C. Dennison , and N. J. Robinson . 2007. “A Periplasmic Iron‐Binding Protein Contributes Toward Inward Copper Supply.” Journal of Biological Chemistry 282, no. 6: 3837–3846. 10.1074/jbc.M609916200. 17148438

[ppl70474-bib-0083] Wegener, K. M. , E. A. Welsh , L. E. Thornton , et al. 2008. “High Sensitivity Proteomics Assisted Discovery of a Novel Operon Involved in the Assembly of Photosystem II, a Membrane Protein Complex.” Journal of Biological Chemistry 283, no. 41: 27829–27837. 10.1074/jbc.M803918200. 18693241

[ppl70474-bib-0084] Zhang, L. F. , H. M. Yang , S. X. Cui , et al. 2009. “Proteomic Analysis of Plasma Membranes of Cyanobacterium *Synechocystis* sp. Strain PCC 6803 in Response to High pH Stress.” Journal of Proteome Research 8, no. 6: 2892–2902. 10.1021/pr900024w. 19351138

