# Supplementary material for: Acclimation of Synechocystis sp. PCC 6803 to Alkaline pH Under Ambient Air
Source: Physiol Plant. 2025 Aug 27;177(5):e70474. doi: 10.1111/ppl.70474 (PMC12382314; doi:10.1111/ppl.70474)
Supplement: Supplementary file 1 — Figure S1: Total protein concentration in a cell lysate. Figure S2: Properties of Synechocystis. Figure S3: Gross O2 evolution of WT and Δ4 strain. Figure S4: Characterization of PSII in the presence of LiCl in cells cultured and measured at pH 7.5. Table S1: Predicted pH and inorganic carbon species in cell‐free BG‐11 medium adjusted to different pH. Table S2: The pH of the BG‐11 used for the cultivations and the pH of the culture supernatant. Table S3: Cell size of Synechocystis. [file PPL-177-e70474-s002.pdf]

Supporting information

Acclimation of *Synechocystis* sp. PCC 6803 to alkaline pH under ambient air

Henna Mustila, Michal Hubáček, Dorota Muth-Pawlak, Yagut Allahverdiyeva

Department of Life Technologies, University of Turku, FI-20014 Turku, Finland

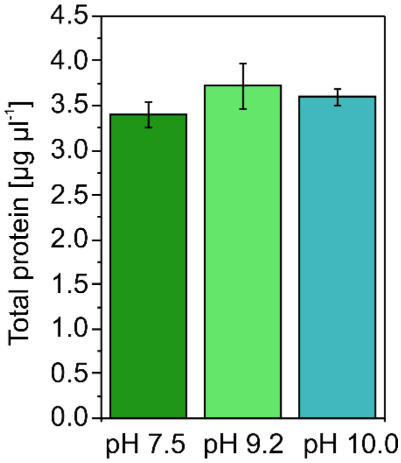

Figure S1. Total protein concentration in a cell lysate. Protein yield from 10 ml of *Synechocystis* wild type cells harvested at OD_{750}»0.9 at various growth pH.

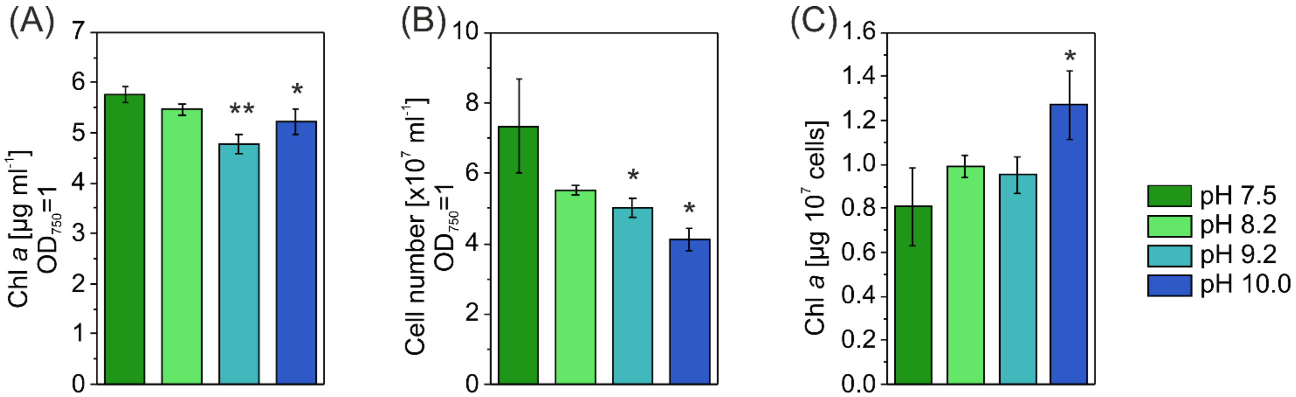

Figure S2. Properties of *Synechocystis*. Chl *a* content (A), the number of cells in 1 ml when the OD_{750} is 1 (B) and the Chl *a* amount per 10^7 cells (C) determined after 4 days cultivation at the corresponding pH. Error bars are standard deviations from 3-4 biologically independent experiments. The asterisks represent significant differences between cells cultivated in medium with pH 7.5 and higher pH as determined by Welch's *t*-test (*$p < 0.05$, **$p < 0.01$, ***$p < 0.001$).

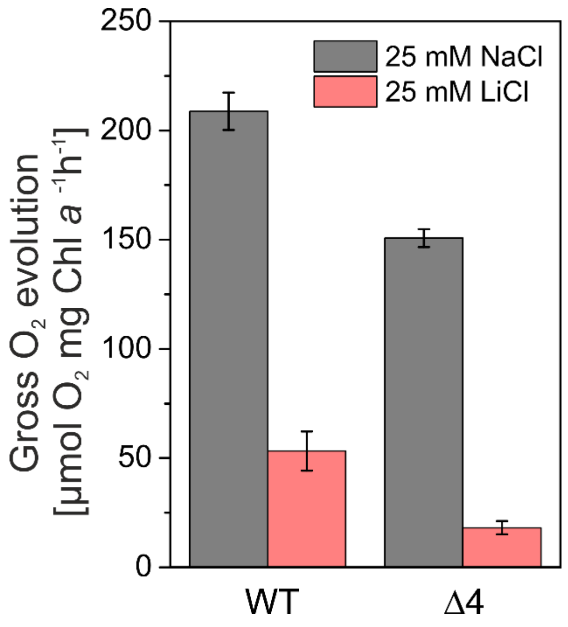

Figure S3. Gross O_2 evolution of WT and Δ4 strain. The WT and Δ4 mutant (Δ*ndhD3/D4/sbtA/cmpA*; Shibata et al. 2002) were grown in BG-11 medium at pH 8.2 under ambient CO_2, as Δ4 strain is unable to grow at higher or lower pH under these conditions. For measurements, cells were suspended in Na^+ free BG-11 medium buffered at pH 10.0. Cells were adjusted to a Chl *a* concentration of 10 µg ml^{-1} and dark adapted for 5 min. O_2 evolution was measured under 500 µmol photons $m^{-2}s^{-1}$ of white actinic light using a Membrane-inlet mass spectrometry. When indicated, 25 mM NaCl or 25 mM LiCl was added prior the dark incubation. Error bars indicate standard deviations (n = 2-3).

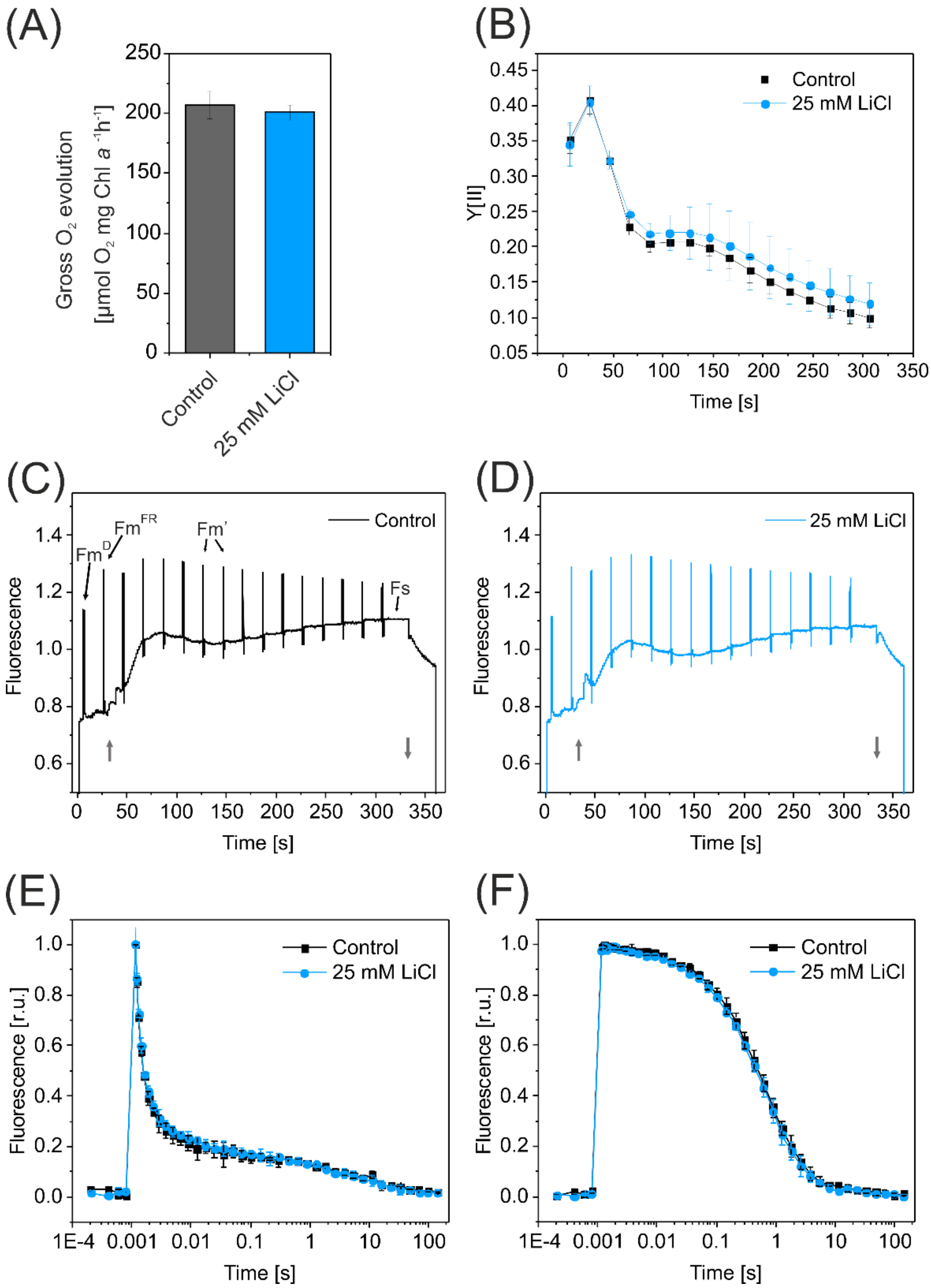

Figure S4. Characterization of PSII in the presence of LiCl in cells cultured and measured at pH 7.5. Gross O_2 evolution rate (A), Y(II) (B), Chl *a* fluorescence induction (C, D) and flash-induced fluorescence yield curves (E, F) were monitored from *Synechosystis*. Cells were grown for 3 days under 50 µmol photons $m^{-2}s^{-1}$ in BG-11 medium buffered with 20 mM Hepes-KOH (pH 7.5). Cells were adjusted to a Chl *a* concentration of 10 µg ml^{-1} (A, B, C, D) or 5 µg ml^{-1} (E, F) in fresh BG-11 (pH 7.5) and dark adapted for 10 min (B, C, D) or 5 min (A, E, F). For gross O_2 measurements, 1.5 mM $KHCO_3$ was added prior recording. When indicated, 25 mM LiCl was added prior the dark incubation. For Chl *a* fluorescence induction curves, background actinic light (58 µmol photons $m^{-2}s^{-1}$) was switched on and off (up and down grey arrow, respectively). Q_A^- reoxidation was monitored from dark-adapted in the absence (E) and in the presence (F) of 20 µM DCMU. For the flash-induced fluorescence decay experiment, the F^0 and F^m values were normalized to 0 and 1, respectively. r.u., Relative units. Error bars indicate standard deviations (n = 3).

Table S1. Predicted pH and inorganic carbon species in cell-free BG-11 medium adjusted to different pH.

pH	TIC (mM)	$[HCO_3^-]$ (mM)	$[CO_3^{2-}]$ (mM)	$[CO_2]$ (mM)	$[HCO_3^-]$ (%)	$[CO_3^{2-}]$ (%)	$[CO_2]$ (%)
7.5	0.18	0.17	0.0004	0.012	93%	0.2 %	7%
8.2	0.86	0.84	0.0094	0.012	98%	1.1 %	1%
9.2	9.3	8.4	0.94	0.012	90%	10%	0.1 %
10	90.5	53.0	37.5	0.012	59%	41%	0.01%

The medium is assumed to reach equilibrium with air level, 0.04%, CO_2. Parameters used for calculation of predicted values (K_H = 0.02964 M atm^{-1}, pK_{a1} = 6.35, pK_{a2} = 10.15) were adapted from Carrasquer-Alvarez et al. 2025. TIC = Total inorganic carbon.

Table S2. The pH of the BG-11 used for the cultivations and the pH of the culture supernatant. The pH of the supernatant was measured when the experimental cultures reached the OD_{750} ≈ 0.9.

Buffer	BG-11, pH	Supernatant when cells harvested, pH
20 mM Hepes-NaOH	7.5	7.63±0.01
20 mM TES-KOH	8.2	8.47±0.02
20 mM CHES-KOH	9.2	9.39±0.02
20 mM CHES-KOH	10.0	10.13±0.02

Table S3. Cell size of *Synechocystis*. Cell diameter of the cells was determined by microscopy and brightfield image analysis with a Cellometer Auto X4 (Nexcelom Bioscience) from cells grown at different pH for 3 and 4 days. The averages and standard deviations were calculated based on three to five biologically independent experiments.

pH	Day 3 (µm)	Day 4 (µm)
7.5	2.0±0.3	2.0±0.3
8.2	2.1±0.2	1.9±0.2
9.2	2.1±0.2	2.0±0.2
10.0	2.0±0.2	2.2±0.2

References

Carrasquer-Alvarez, E., Hoffmann, U.A., Geissler, A.S. et al. (2025). Photosynthesis in *Synechocystis* sp. PCC 6803 is not optimally regulated under very high CO_2. *Applied Microbiology and Biotechnology* 109(1), 33.

Shibata, M., Katoh, H., Sonoda, M., Ohkawa, H., Shimoyama, M., Fukuzawa, H., Kaplan, A., & Ogawa, T. (2002). Genes essential to sodium-dependent bicarbonate transport in cyanobacteria: Function and phylogenetic analysis. *Journal of Biological Chemistry*, 277(21), 18658–18664.
